# Bifurcation analysis of two coupled Jansen-Rit neural mass models

**DOI:** 10.1371/journal.pone.0192842

**Published:** 2018-03-27

**Authors:** Saeed Ahmadizadeh, Philippa J. Karoly, Dragan Nešić, David B. Grayden, Mark J. Cook, Daniel Soudry, Dean R. Freestone

**Affiliations:** 1 Department of Electrical and Electronic Engineering, The University of Melbourne, Melbourne, VIC, Australia; 2 Department of Biomedical Engineering, The University of Melbourne, Melbourne, VIC, Australia; 3 Department of Medicine, St. Vincent’s Hospital Melbourne, The University of Melbourne, Melbourne, VIC, Australia; 4 Department of Statistics, Columbia University, New York, New York, 10027, United States of America; 5 Centre for Neural Engineering, The University of Melbourne, Melbourne, VIC, Australia; Georgia State University, UNITED STATES

## Abstract

We investigate how changes in network structure can lead to pathological oscillations similar to those observed in epileptic brain. Specifically, we conduct a bifurcation analysis of a network of two Jansen-Rit neural mass models, representing two cortical regions, to investigate different aspects of its behavior with respect to changes in the input and interconnection gains. The bifurcation diagrams, along with simulated EEG time series, exhibit diverse behaviors when varying the input, coupling strength, and network structure. We show that this simple network of neural mass models can generate various oscillatory activities, including delta wave activity, which has not been previously reported through analysis of a single Jansen-Rit neural mass model. Our analysis shows that spike-wave discharges can occur in a cortical region as a result of input changes in the other region, which may have important implications for epilepsy treatment. The bifurcation analysis is related to clinical data in two case studies.

## Introduction

Epilepsy is regarded as the second most common neurological disease after stroke. The hallmark of epilepsy is recurrent unprovoked seizures, during which a network of the brain is hyper-excitable [1]. Medication is the main treatment for controlling epilepsy. However, approximately 30% of patients are not well treated by anti-epileptic drugs and suffer from recurring seizures [2]. Epilepsy surgery is a treatment option for patients whose seizures continue despite pharmacological interventions. However, surgical intervention is not viable for all patients due to the risks involved in the removal of brain tissue [3]. Hence, there is a strong research effort directed towards alternative methods to control seizures. In order to develop new robust therapies, there is a need to understand the mechanisms that lead to seizures. This has proven to be a difficult problem to unravel from an experimental point of view. Therefore, computational modeling studies are an alternative to understand epilepsy at a network level and generate new hypotheses regarding the basic mechanisms that lead to seizures.

Over the past sixty years, computational neural modeling has contributed to the development of theory that explains brain dynamics at different spatiotemporal scales. Microscopic models, such as those of [4] and [5], describe single neuron dynamics. Mesoscopic neural mass models have also been developed in parallel to the microscopic models, with notable early contributions from [6, 7], and [8]. Mesoscopic, neural mass, or neural field models describe the averaged activity of cortical ensembles. Modeling at the mesoscopic scale is particularly important for epilepsy, as this is the scale observed through clinical electroencephalographic (EEG) and intracranial EEG recordings.

There are numerous studies that have used neural mass models to study epilepsy. The models generate hypotheses regarding the mechanisms that underlie the transitions from normal brain activity to seizures. For example, [9] used a model proposed by [10] to replicate alpha and epileptic-like activity by changing the model parameters. The same group also developed a multi-region model to study the effect of changing long-range connectivity [9]. They observed that, for high interconnection gains, all regions showed synchronous behavior that mimicked electrographic seizure recordings. These results motivated other researchers to further develop and investigate neural mass models to reproduce a wider range of observable brain dynamics (see [11–14] for more information).

Recently, [15] investigated the effects of network structure on seizure spread in a four-region network through computer simulation. Their results demonstrated that seizure spread from an onset region was highly dependent on the structure of the network. Furthermore, altering the network structure by adding or removing interconnections between regions could preserve or annihilate seizures. They also presented a network structure in which some regions show seizure behavior while the other regions show normal behavior. These results highlight that the configuration of populations in the network significantly affects the initiation and propagation of epileptic seizures. These analyses, based on computer simulations, can be studied more rigorously by tools from control theory [16, 17] and graph theory [18, 19], or by a bifurcation analysis.

Bifurcation analysis enables visualization of the dynamical repertoire of a computational model undergoing parameter variations. For example, a bifurcation analysis will show where a model that is undergoing parameter changes transitions into different types of oscillations. [20] used bifurcation analysis to show how changes in the external input to neural mass models led to alpha-like signals described by a limit cycle and seizure-like output described by an orbit that results from a saddle-node homoclinic bifurcation. More recently, a bifurcation analysis of a neural mass model with variations in a time delay revealed a possible mechanism for the transition from alpha to seizure activity [21]. Understanding how such bifurcations occur is critical in interpreting many high-level brain functions. Using bifurcation analysis, [22] provided evidence that functional connectivity may be increased during seizures.

Previous bifurcation analyses of neural mass models have enabled theoretical and computational studies to reproduce important activity of the brain, providing insights into possible mechanisms underlying transitions between different brain states. However, networked neural mass models have not been widely analyzed in this way. It is well known that network structure has a significant effect on cortical dynamics, such as seizure generation. Therefore, a bifurcation analysis to study the behavior of two interconnected neural mass models is an important step towards understanding how network structure mediates seizure mechanisms. Although bifurcation analyses of networked neural mass models have been previously reported, for instance [23, 24], previous studies used different models such as a Wilson-Cowan neural population. The current study provides a bifurcation analysis of two interconnected Jansen-Rit neural populations, which each consist of three interacting neural populations. Models with three or more populations exhibit a range of dynamics that align with many different observed cortical activities, especially epileptic activities [14]. Bifurcation analysis of two interconnected Jansen-Rit neural populations was recently studied in [25], where the input of the network was fixed to a specific value at which the single neural mass population exhibits the oscillatory behavior. In [25] the effects of changing the interconnection gain were studied by computing the maximal Lyapunov exponent (MLE) for limit cycles. In [26, 27], the authors conducted bifurcation analysis of two interconnected Jansen-Rit neural populations in a region in which the network shows an epileptic behavior. Both inputs and interconnection gains were considered as the bifurcation parameters in [26, 27]. In contrast to aforementioned work, we explored a wide range of network behaviors, rather than focusing on a specific region of the parameter space. By changing the network configuration and external inputs, we found unique behaviours for a coupled network, which were not possible for a single neural mass model. Furthermore, we explored unexpected dynamics of the network that have important implications for epilepsy related surgery. Finally, we demonstrate our analysis is relevant for real world epileptic seizures, by relating the bifurcation diagrams to data using a parameter inference method.

This paper is organized as follows. In Section 1.2, we introduce the multi-region neural mass model that is used in this study. Sections 1.3 to 2.3 present bifurcation analyses for three different settings of inter-connectivity. Section 2.4 relates the estimation results to the bifurcation analyses. Finally, we demonstrate how clinical insights are gained from our new analyses, and discuss future work in Section 3.

## 1 Methods

### 1.1 Ethics statement

The research involving human intracranial EEG data, presented in Section 2.4, was approved by the Human Research Ethics Committee at St. Vincent’s Hospital Melbourne (Low Risk Research 145/13).

### 1.2 Model description

In this section, we briefly present the mathematical representation of a neural mass model that describes a cortical area. We start from a model proposed by [28] that is used in Section 2.4. We explain how this model can be reduced to achieve the well-known model described in previous work [9, 10]. The [28] model contains three parts: pyramidal neurons, excitatory (spiny stellate) neurons, and inhibitory neurons. A pyramidal unit receives input from three sources: distant regions *u*, an excitatory unit *v*_*e*_, and an inhibitory unit *v*_*i*_. The dynamics of the neural mass model are described by the following set of ordinary differential equations [28]:

$$\begin{array}{c} \begin{array}{cc} {\dot{v}}_{e}= & z_{e}\\ {\dot{z}}_{e}= & \alpha_{pe}c_{pe}\zeta_{pe}g(v_{p})-2\zeta_{pe}z_{e}-\zeta_{pe}^{2}v_{e}\\ {\dot{v}}_{i}= & z_{i}\\ {\dot{z}}_{i}= & \alpha_{pi}c_{pi}\zeta_{pi}g(v_{p})-2\zeta_{pi}z_{i}-\zeta_{pi}^{2}v_{i}\\ {\dot{v}}_{p1}= & z_{p1}\\ {\dot{z}}_{p1}= & \alpha_{ep}c_{ep}\zeta_{ep}g(v_{e})-2\zeta_{ep}z_{p1}-\zeta_{ep}^{2}v_{p1}\\ {\dot{v}}_{p2}= & z_{p2}\\ {\dot{z}}_{p2}= & \alpha_{ip}c_{ip}\zeta_{ip}g(v_{i})-2\zeta_{ip}z_{p2}-\zeta_{ip}^{2}v_{p2}\\ {\dot{v}}_{p3}= & z_{p3}\\ {\dot{z}}_{p3}= & \alpha_{up}c_{up}\zeta_{up}u-2\zeta_{up}z_{p3}-\zeta_{up}^{2}v_{p3}, \end{array} \end{array}$$ (1)

where the post-synaptic potential, denoted by *v*_*n*_, is the deviation of the membrane from the resting potential, *α*_*mn*_ is the gain for the post-synaptic response kernel, *c*_*mn*_ is the number of connections between populations, and *ζ*_*mn*_ is the reciprocal of the synaptic/membrane time constant. The index *n* (post-synaptic) may represent the pyramidal (*p*), excitatory interneuron (spiny stellate) (*e*), or inhibitory interneuron (*i*) populations. The parameter *u* describes the external input firing rate. *v*_*p*1_, *v*_*p*2_, *v*_*p*3_ (mV) are post-synaptic potential on the pyramidal cell induced by excitatory feedback, inhibitory feedback and external input, respectively. The post-synaptic potential of the pyramidal cell is then defined as *v*_*p*_ = *v*_*p*1_ − *v*_*p*2_ + *v*_*p*3_. The sigmoid function, *g*(*v*_*m*_), characterizes internal firing rates as a function of the pre-synaptic (subscript *m*) membrane potential, defined by

$$\begin{array}{c} g\left(v\right)=\frac{2e_{0}}{1+\exp(r(v_{th}-v))}, \end{array}$$ (2)

where *r* defines the slope of the sigmoid, *v*_*th*_ is the mean firing threshold, and 2*e*_0_ is the maximum firing rate.

In order to achieve the model in [9, 10], it is first assumed that the following set of equalities holds on excitatory gains and time constants, *α*_*pe*_ = *α*_*pi*_ = *α*_*ep*_ = *α*_*up*_ ≜ *α*_*e*_, *ζ*_*pe*_ = *ζ*_*pi*_ = *ζ*_*ep*_ = *ζ*_*up*_ ≜ *ζ*_*e*_, *α*_*ip*_ ≜ *α*_*i*_, *ζ*_*ip*_ ≜ *ζ*_*i*_. These assumptions imply that the internal mathematical models of excitatory and inhibitory neurons are the same; however, their influence on post-synaptic potential of the pyramidal cell are different. Furthermore, the same mathematical formulation is used to model the influence of input *u* and excitatory feedback *v*_*p*1_ on the pyramidal cell. Therefore, we can define a new variable that incorporates the influence of *u* and *v*_*p*1_, leading to

$$\begin{array}{l} v_{1}≜v_{p1}+v_{p3},\\ v_{2}≜v_{p2},\\ z_{1}≜z_{p1}+z_{p3},\\ z_{2}≜z_{p2}. \end{array}$$

Given the above definition, the post-synaptic potential of the pyramidal cell can be written as *v*_*p*_ = *v*_1_ − *v*_2_. Furthermore, it is supposed that the co-activation of spiny stellate and inhibitory cells are proportional and mathematically described as,

$$\begin{array}{c} \begin{array}{c} v_{0}≜\frac{v_{i}}{c_{pi}}=\frac{v_{e}}{c_{pe}}, \end{array}\\ \begin{array}{c} z_{0}≜\frac{z_{i}}{c_{pi}}=\frac{z_{e}}{c_{pe}}. \end{array} \end{array}$$

It is also assumed that the number of connections between the input and the pyramidal cells is equal to one, i.e. *c*_*up*_ = 1. It should be pointed out that this assumption is not conservative mathematically since we consider $\tilde{u}≜c_{up}u$ as a new input for Eq (1). Considering all aforementioned assumptions, the tenth-order system in Eq (1) is reduced to the sixth-order state-space model,

$$\begin{array}{c} \begin{array}{cc} {\dot{v}}_{0} & =z_{0}\\ {\dot{z}}_{0} & =\alpha_{e}\zeta_{e}g\left(v_{1}-v_{2}\right)-2\zeta_{e}z_{0}-{\zeta_{e}}^{2}v_{0}\\ {\dot{v}}_{1} & =z_{1}\\ {\dot{z}}_{1} & =\alpha_{e}\zeta_{e}(u+c_{ep}g\left(c_{pe}v_{0}\right))-2\zeta_{e}z_{1}-{\zeta_{e}}^{2}v_{1}\\ {\dot{v}}_{2} & =z_{2}\\ {\dot{z}}_{2} & =\alpha_{i}\zeta_{i}c_{ip}g\left(c_{pi}v_{0}\right)-2\zeta_{i}z_{2}-{\zeta_{i}}^{2}v_{2}. \end{array} \end{array}$$ (3)

**Fig 1:**
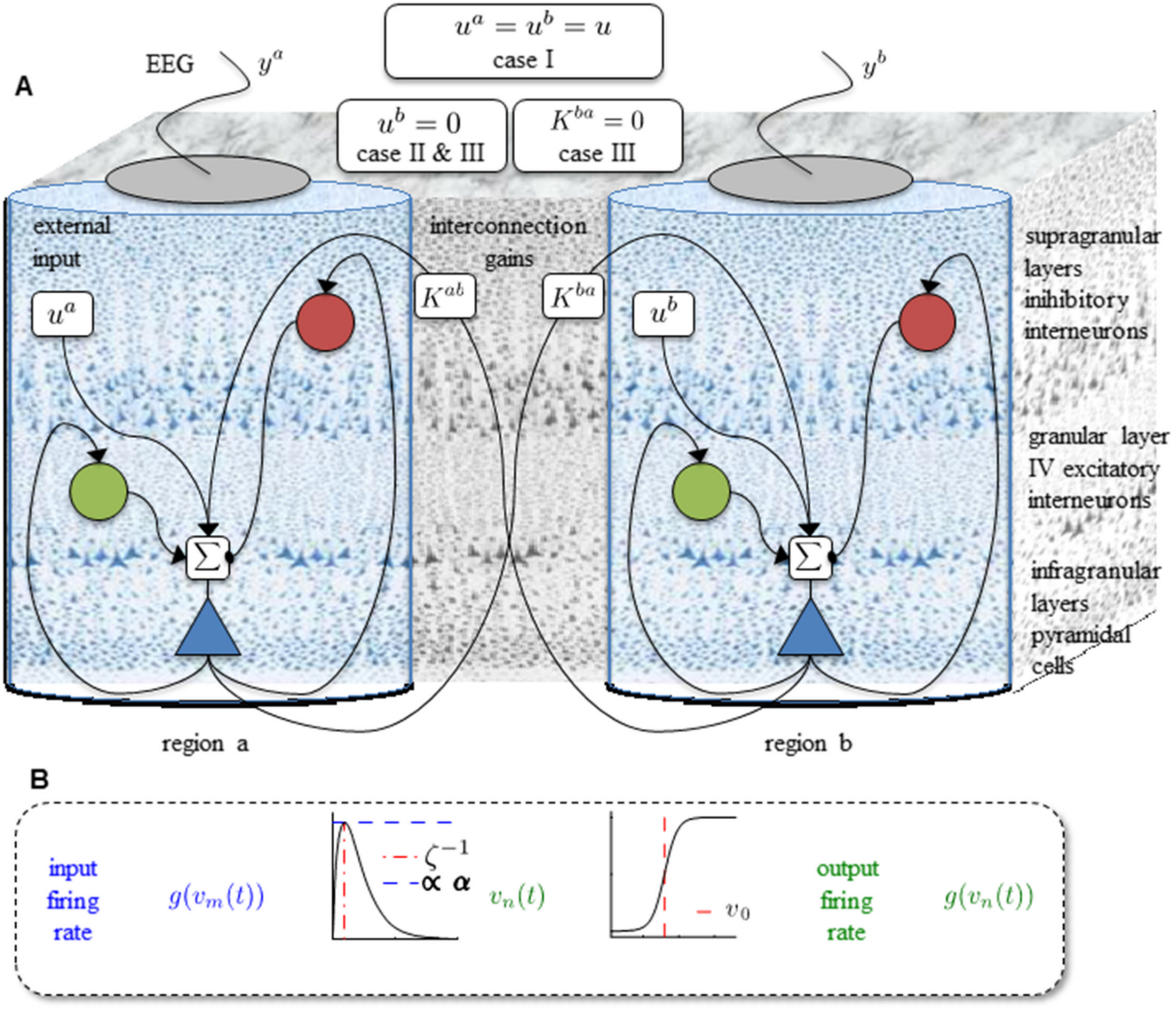
The schematic diagram of network of Jansen’s model and three underlying cases. A. The schematic diagram of the neural mass model for two cortical regions described by Eq 4. B. Elements of a neural mass, showing the synaptic kernel on the left and the sigmoidal nonlinearity on the right.

Eq 3 describes the reduced model of single neural mass model. In order to interconnect the reduced neural mass models and construct a network, it is assumed that the pyramidal unit also receives input from neighboring regions that is added to the external input *u*. In this case, the neural mass model network with *N* regions is described by [9, 10]

$$\begin{array}{c} \begin{array}{cc} {\dot{v}}_{0}^{j} & =z_{0}^{j}\\ {\dot{z}}_{0}^{j} & =\alpha_{e}^{j}\zeta_{e}^{j}g\left(v_{1}^{j}-v_{2}^{j}\right)-2\zeta_{e}^{j}z_{0}^{j}-{\zeta_{e}^{j}}^{2}v_{0}^{j}\\ {\dot{v}}_{1}^{j} & =z_{1}^{j}\\ {\dot{z}}_{1}^{j} & =\alpha_{e}^{j}\zeta_{e}^{j}(u^{j}+c_{ep}^{j}g\left(c_{pe}^{j}v_{0}^{j}\right)+\sum_{l=1,l\neq j}^{N}K^{j,l}v_{3}^{l})-2\zeta_{e}^{j}z_{1}^{j}-{\zeta_{e}^{j}}^{2}v_{1}^{j}\\ {\dot{v}}_{2}^{j} & =z_{2}^{j}\\ {\dot{z}}_{2}^{j} & =\alpha_{i}^{j}\zeta_{i}^{j}c_{ip}^{j}g\left(c_{pi}^{j}v_{0}^{j}\right)-2\zeta_{i}^{j}z_{2}^{j}-{\zeta_{i}^{j}}^{2}v_{2}^{j}\\ {\dot{v}}_{3}^{j} & =z_{3}^{j}\\ {\dot{z}}_{3}^{j} & =\alpha_{e}^{j}\zeta_{d}^{j}g\left(v_{1}^{j}-v_{2}^{j}\right)-2\zeta_{d}^{j}z_{3}^{j}-{\zeta_{d}^{j}}^{2}v_{3}^{j}, \end{array} \end{array}$$ (4)

where superscript *j* = 1, …, *N* indexes the neural mass in region *j*. The parameters $\alpha_{e}^{j},\alpha_{i}^{j},\zeta_{e}^{j},\zeta_{i}^{j},\zeta_{d}^{j}$ are considered known. The two state variables *v*_3_ and *z*_3_ are used to interconnect region *j* to the other regions in the network. The effect of external regions on local dynamics is parametrized by the coupling gain *K*^*j*,*l*^ ≥ 0 (mV^−1^s^−1^) and coupling outputs $v_{3}^{l}$ [9, 10]. Note that *K*^*i*,*i*^ = 0, *i* = 1, …, *n*. A schematic diagram of a two-region network is depicted in Fig 1.

The model (4) implies that each region *j* shows different behaviors depending on the region parameters, external inputs *u*^*j*^(*t*) (s^−1^) and coupling gains. The complexity of the model is increased dramatically for a network with a large number of regions. Even for a network with two regions, it is difficult to analyze the effects of variations of parameters and coupling gains. In this manuscript, we consider a network with *N* = 2 regions, region *a* and region *b*, and provide a rigorous analysis. The model parameters and their interpretation are given in Table 1 (also see [9]).

**Table 1:** The parameters of model (4), used in Jansen and Rit’s original model [9].

Parameter	Description	Value
*α*_*e*_, *α*_*i*_	Average gain of excitatory (e) and inhibitory (i) synaptic gains	*α*_*e*_ = 3.25 mV, *α*_*i*_ = 22 mV
$\frac{1}{\zeta_{e}}$, $\frac{1}{\zeta_{i}}$, $\frac{1}{\zeta_{d}}$	Average time constant of post-synaptic potential. *d* is the connection between regions.	*ζ*_*e*_ = 100 s^−1^, *ζ*_*i*_ = 50 s^−1^, *ζ*_*d*_ = 33 s^−1^
*c*_*pe*_, *c*_*ep*_, *c*_*pi*_, *c*_*ip*_	Average number of synaptic contacts of excitatory and inhibitory connection	*c*_*pe*_ = *c*, *c*_*ep*_ = 0.8*c*, *c*_*pi*_ = 0.25*c*, *c*_*ip*_ = 0.25*c* with *c* = 135
*v*_*th*_, *e*_0_, *r*	Threshold, half of the maximum output, and slope of sigmoid function *g*(*v*)	*v*_*th*_ = 6 mV, *e*_0_ = 2.5 s^−1^, *r* = 0.56 mV^−1^

We now state the assumptions that are required for further analysis. The first assumption is that the local parameters of the two regions are identical, and changes in the network behavior result from a varying input. This assumption implies that these two regions belong to the same cortical area. For Sections 2.1 and 2.2, we will make a second assumption that the coupling gains between the two regions are symmetric; i.e., *K*^1,2^ = *K*^2,1^ = *K*. The second assumption is relaxed in Section 2.3. Although the assumptions limit the generality of the results, the networks shows very complicated behavior when the coupling gain is varied and valuable insights are gained. The assumptions are required to gain these insights and similar approaches have been used in previous studies [9, 15].

Three cases are analyzed (see Fig 1). In case I, the same input is applied to both regions. This structure can be seen as a network of two regions that are located near each other and receive common input. These two regions are involved in the same function; i.e., the same input and the same hierarchical level. In case II, we assume that only region *a* receives input, representing two regions that could be in same area with the same parameters, but with different levels of hierarchy. In case III, region *a* receives input and the feedback from region *b* is removed. In Section 2.2 and 2.3, we will point out that this change in the structure of the network induces interesting changes in the dynamics.

### 1.3 Equilibria

In order to start the bifurcation analysis, the first step is to find the equilibria of the network by setting the left hand side of (4) to zero for *j* = *a*, *b*. This leads to the following set of equations:

$$\begin{array}{c} \begin{array}{cccc} v_{0}^{a} & =\frac{\alpha_{e}}{\zeta_{e}}g(v_{1}^{a}-v_{2}^{a}), & & z_{0}^{a}=0,\\ v_{1}^{a} & =\frac{\alpha_{e}}{\zeta_{e}}(u^{a}+c_{ep}g(c_{pe}v_{0}^{a})+K^{ab}v_{6}^{b}), & & z_{1}^{a}=0,\\ v_{2}^{a} & =\frac{\alpha_{i}}{\zeta_{i}}c_{ip}g(c_{pi}v_{0}^{a}), & & z_{2}^{a}=0,\\ v_{3}^{a} & =\frac{\alpha_{e}}{\zeta_{d}}g(v_{1}^{a}-v_{2}^{a}), & & z_{3}^{a}=0. \end{array} \end{array}$$ (5)

$$\begin{array}{c} \begin{array}{cccc} v_{0}^{b} & =\frac{\alpha_{e}}{\zeta_{e}}g(v_{1}^{b}-v_{2}^{b}), & & z_{0}^{b}=0,\\ v_{1}^{b} & =\frac{\alpha_{e}}{\zeta_{e}}(u^{b}+c_{ep}g(c_{pe}v_{0}^{b})+K^{ba}v_{6}^{a}), & & z_{1}^{b}=0,\\ v_{2}^{b} & =\frac{\alpha_{i}}{\zeta_{i}}c_{ip}g(c_{pi}v_{0}^{b}), & & z_{2}^{b}=0,\\ v_{3}^{b} & =\frac{\alpha_{e}}{\zeta_{d}}g(v_{1}^{b}-v_{2}^{b}), & & z_{3}^{b}=0. \end{array} \end{array}$$ (6)

We define the EEG signal corresponding to region a and region b as $y^{a}≔v_{1}^{a}-v_{2}^{a}$ and $y^{b}≔v_{1}^{b}-v_{2}^{b}$ [9]. Now, from (5) and (6), we can write the equations describing the EEG at equilibrium as [25]

$$\begin{array}{c} \begin{array}{cc} y^{a}= & \frac{\alpha_{e}}{\zeta_{e}}u^{a}+\frac{\alpha_{e}}{\zeta_{e}}c_{ep}g(\frac{\alpha_{e}}{\zeta_{e}}c_{pe}g\left(y^{a}\right))-\frac{\alpha_{i}}{\zeta_{i}}c_{ip}g(\frac{\alpha_{e}}{\zeta_{e}}c_{pi}g\left(y^{a}\right))+\frac{\alpha_{e}^{2}}{\zeta_{e}\zeta_{d}}K^{ab}g\left(y^{b}\right),\\ y^{b}= & \frac{\alpha_{e}}{\zeta_{e}}u^{b}+\frac{\alpha_{e}}{\zeta_{e}}c_{ep}g(\frac{\alpha_{e}}{\zeta_{e}}c_{pe}g\left(y^{b}\right))-\frac{\alpha_{i}}{\zeta_{i}}c_{ip}g(\frac{\alpha_{e}}{\zeta_{e}}c_{pi}g\left(y^{b}\right))+\frac{\alpha_{e}^{2}}{\zeta_{e}\zeta_{d}}K^{ba}g(y^{a}). \end{array} \end{array}$$ (7)

Since (7) is implicit in terms of *u*^*a*^, *u*^*b*^, *K*^*ab*^ and *K*^*ba*^, a computational approach is utilized in which the values of *u*^*a*^, *u*^*b*^, *K*^*ab*^ and *K*^*ba*^ are considered to be fixed, and the values of *y*^*a*^ and *y*^*b*^ are obtained subsequently. Then, the equilibria of the network corresponding to those fixed values can be determined from (5) and (6). The goal of the bifurcation analysis is to analyze the behavior of the underlying network arising around equilibria as parameters of the network are varied.

## 2 Results

### 2.1 Case I: Bifurcation analysis with a common input

In case I, the applied inputs and interconnection gains are considered to be same for both regions, i.e., *u*^*a*^ = *u*^*b*^ = *u* and *K*^*ab*^ = *K*^*ba*^ = *K*. We consider *u* as the bifurcation parameter that changes continuously, and consider discrete interconnection gains *K* = 25, 50, 100, 150. Considering both the input *u* and the interconnection gain *K* as continuous bifurcation parameters provides a more comprehensive analysis of the underlying networks, but is beyond the scope of this paper.

We categorize the equilibria of the network into two groups. The first group contains the set of equilibria, called symmetric equilibria, that are equal; i.e., *y*^*a*^ = *y*^*b*^ = *y*^*s*^. This set of equilibria results from the symmetrical structure of network, which can be observed from both Fig 1 and Eq (7). The symmetry makes it possible to rewrite Eq (7) to

$$\begin{array}{c} \begin{array}{cc} y^{s}= & \frac{\alpha_{e}}{\zeta_{e}}u+\frac{\alpha_{e}}{\zeta_{e}}c_{ep}g(\frac{\alpha_{e}}{\zeta_{e}}c_{pe}g\left(y^{s}\right))-\frac{\alpha_{i}}{\zeta_{i}}c_{ip}g(\frac{\alpha_{e}}{\zeta_{e}}c_{pi}g\left(y^{s}\right))+\frac{\alpha_{e}^{2}}{\zeta_{e}\zeta_{d}}Kg\left(y^{s}\right), \end{array} \end{array}$$ (8)

which is used to compute the symmetric equilibria. The second group of equilibria correspond to the asymmetric solutions, which are unequal. The asymmetric equilibria are computed using Eq (7).

Note that both Eqs (7) and (8) are nonlinear, so it is not possible to find explicit expressions for *y*^*a*^ and *y*^*b*^ in terms of *u* and *K*. Therefore, we utilize a numerical approach to find the solutions by changing the value of *y*^*s*^ ∈ (−3.5, 12) in Eq (8) and then calculating the value of the corresponding input *u*. The asymmetric equilibria are computed using the feature of the CL-MATCONT package [29], that exploits the continuity of solutions with respect to the variation of *u*.

#### 2.1.1 Bifurcation analysis with weak coupling (*K* = *25*)

**Fig 2:**
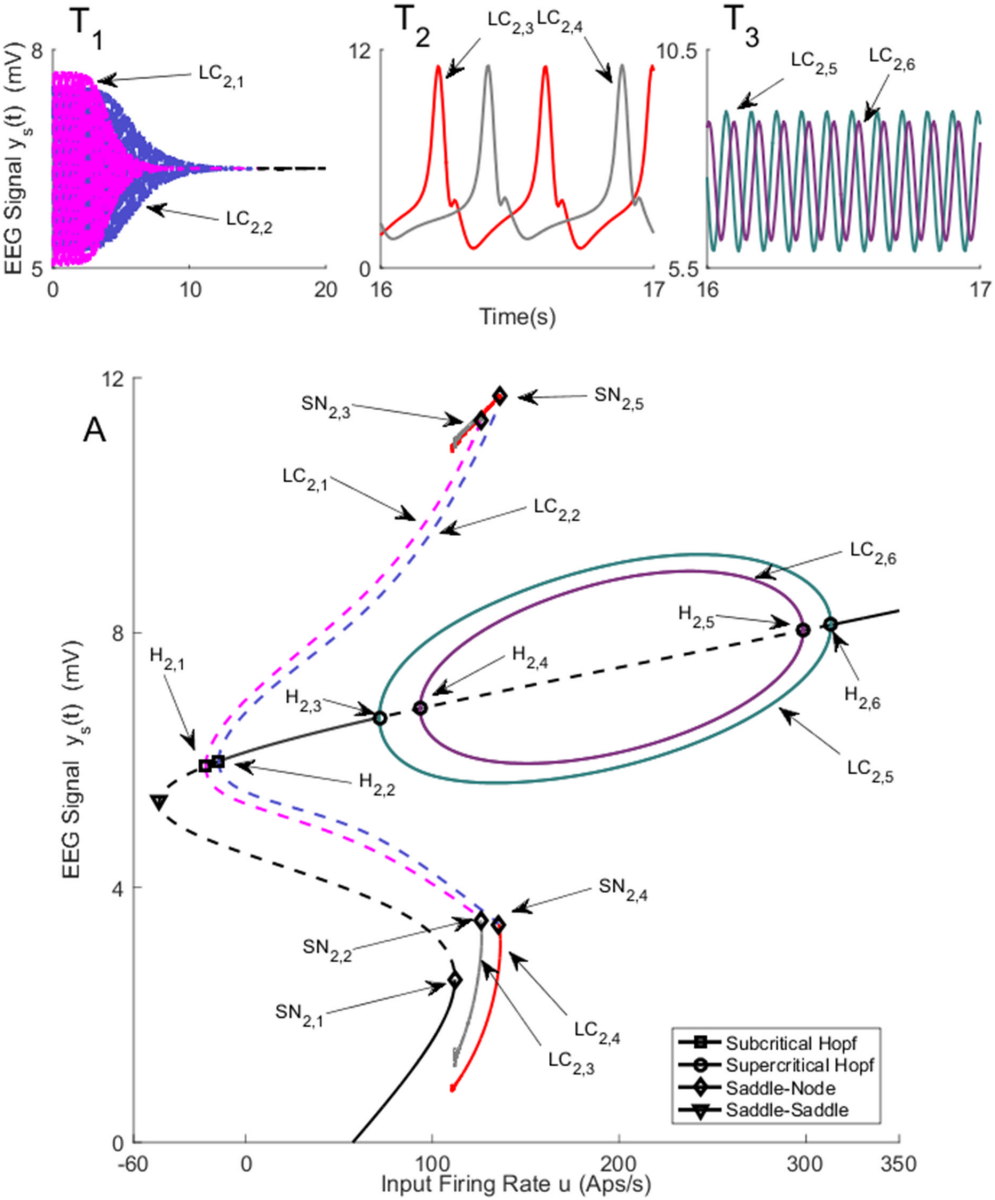
Bifurcation diagram for the symmetric solution for case I with coupling gain *K* = 25. The time series in Panels T_1_ − T_3_) show the EEG associated with each bifurcation using the same color. The solid black lines show stable fixed points, the solid colored lines show stable oscillatory behavior and the dashed lines show unstable fixed points and unstable oscillations. The initial conditions and the corresponding values of the input *u* for all times series are provided in Appendix B.

Two separate bifurcation analyses were conducted corresponding to the symmetric and asymmetric solutions to the equilibria. The equilibria that correspond to the symmetric solution are shown in Fig 2A. The values of *u* for all bifurcation points for symmetric case are presented in Table 2. In all figures presented in this paper, the solid black lines represent the stable equilibria; i.e, all eigenvalues of the Jacobian matrix have negative real parts, and the black dashed lines show unstable equilibria. Fig 2A shows two subcritical Hopf bifurcations H_2,1_ and H_2,2_ that occur where the input, *u* = −14.46 or *u* = −21.43. For a single region neural mass model, there is only one corresponding subcritical Hopf bifurcation [20]. These two subcritical Hopf bifurcation lead to the presence of two limit cycles LC_2,1_ and LC_2,2_. The simulated EEG signals corresponding to each limit cycle are shown in Fig 2T_1_ (the initial conditions and the corresponding values of the input *u* for all times series are provided in Appendix B). Since the limit cycles are unstable, they repel nearby trajectories and, consequently, the trajectories are attracted by the stable equilibria.

**Table 2:** The values of input *u* at bifurcation points in Fig 2.

Bifurcation	H_2,1_	H_2,2_	H_2,3_	H_2,4_	H_2,5_	H_2,6_	SN_2,1_	SN_2,2_ & SN_2,3_	SN_2,4_ & SN_2,5_
Values of *u*	-21.43	-14.46	71.56	93.4	298.6	313.4	112	125.7	135.4

Fig 2A also shows a saddle-node homoclinic bifurcation, indicated by SN_2,1_, when the input *u* = 110.5. The saddle-node homoclinic bifurcation leads to the appearance of two orbits. We point out that a Shlinkov saddle-node can have more than one homoclinic orbit simultaneously if the dimension of the underlying system (number of states) is strictly larger than 2. An example of Shlinkov saddle-node with a pair of the homoclinic orbits is reported in the modified Morioka-Simizu model [30]. More information can also be found in http://www.scholarpedia.org/article/Shilnikov_saddle-node_bifurcation. In our study, the dimension of the system is 16. LC_2,3_ and LC_2,4_ (see Appendix A for details of Shilnikov saddle-node homoclinic bifurcation detection) that generate epileptic-like spike and wave discharges, as seen in Fig 2T_2_. The two types of spike and wave discharges have the same frequency as each other, but different amplitudes. The orbit LC_2,3_, which is plotted in grey, terminates when the input *u* exceeds 125.7. This termination occurs at SN_2,2_ and SN_2,3_, which is due to a collision of the stable cycle LC_2,3_ with the unstable limit cycle LC_2,1_ originating from the subcritical Hopf bifurcation H_2,1_. Similarly, the orbit LC_2,4_ plotted in red, collides at SN_2,4_ and SN_2,5_ (*u* = 136.4) with the unstable limit cycle LC_2,2_ originating from the subcritical Hopf bifurcation H_2,2_.

A supercritical Hopf bifurcation H_2,3_ occurs when the input is increased above *u* = 71.56. The stable equilibrium point becomes unstable resulting from the complex eigenvalues of the Jacobian matrix crossing the imaginary axis. This Hopf bifurcation gives rise to a stable limit cycle LC_2_. Another two complex eigenvalues cross the imaginary axis when the input reaches *u* = 93.46 resulting in another supercritical Hopf bifurcation H_2,4_. It should be noted that the equilibrium point remains unstable since the Jacobian matrix has eigenvalues with positive real part. In multi-dimensional systems, Hopf bifurcation occurs if a pair of complex eigenvalues crosses the imaginary axis while the rest of eigenvalues can have positive or negative real parts. The type of bifurcation (supercritical or subcritical) is determined by computing the first Lyapunov coefficient (see [31, Chapter 5]). These two Hopf bifurcations lead to the appearance of two stable limit cycles LC_2_ and LC_3_. These two limit cycles disappear when the input exceeds 298.6 and 313.4. Fig 2T_3_ shows the alpha rhythm-like EEG for stable limit cycles with a frequency of approximately 10Hz. The two alpha-like oscillations have slightly different amplitudes and frequencies.

**Fig 3:**
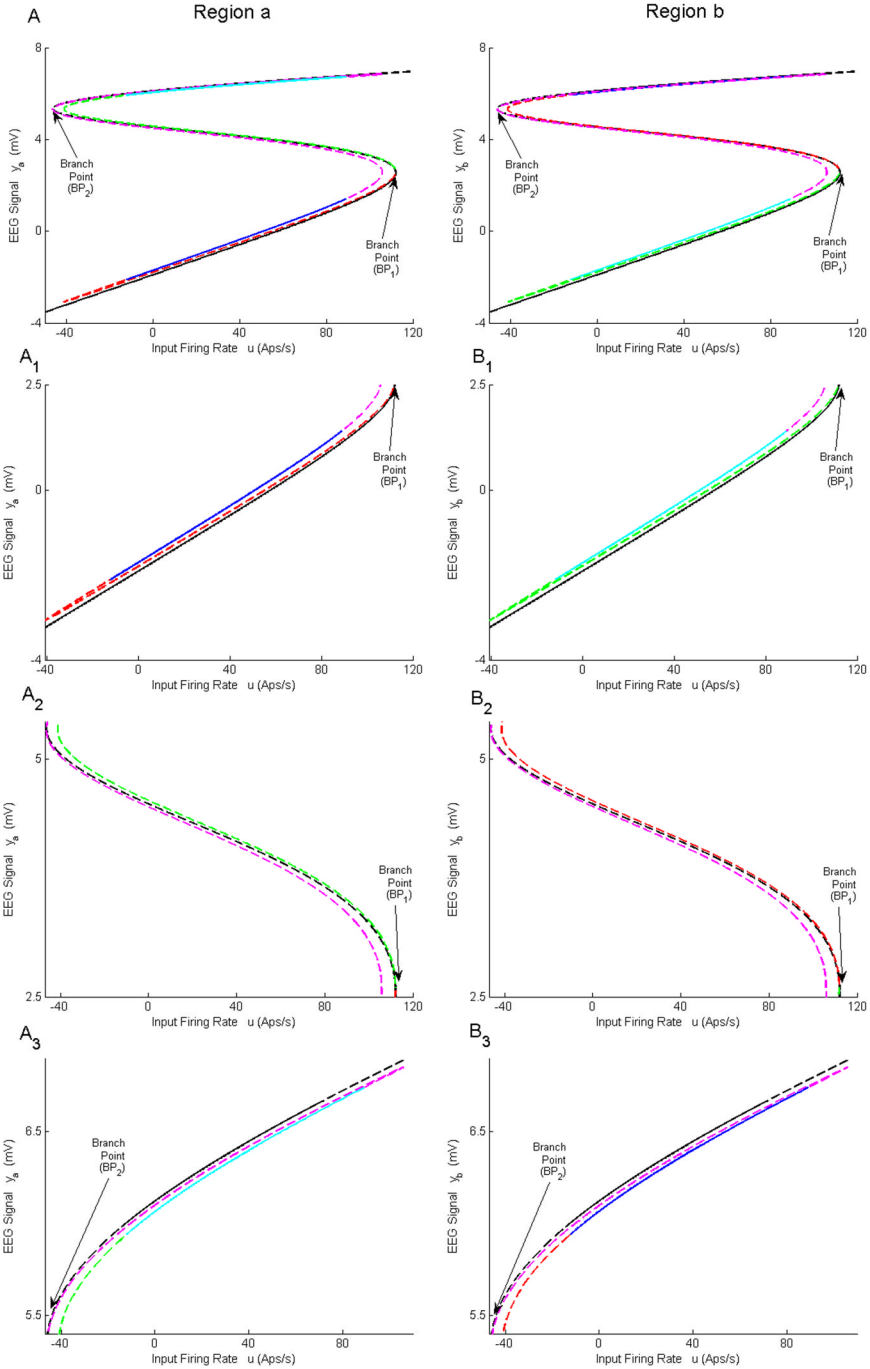
Equilibria curves for asymmetric solutions of case I with coupling gain *K* = 25. A) and B) Second branch of equilibria for regions *a* and *b*, respectively. A_1_-A_3_) Magnified parts from panel in A) corresponding to the bottom, the middle and the upper parts. B_1_-B_3_) Magnified parts from panel in B) corresponding to the lower, the middle and the top parts. The black lines correspond to the symmetric solutions; the red, blue, green, cyan and magenta lines correspond to asymmetric solutions. The equilibria for *y*^*a*^ and *y*^*b*^ are shown with the same color and line style. For example, an equilibrium point with blue solid-line in Panel A_1_) corresponds to an equilibrium point with blue solid-line in Panel B_3_).

During continuation, two branch points BP_1_ and BP_2_ were detected on the symmetric equilibria curve. At these points, other branches of equilibria arise that correspond to the asymmetric solution, and are depicted in Fig 3A and 3B for region *a* and region *b*, respectively. Fig 3A_1_, 3A_2_ and 3A_3_ (Fig 3B_1_, 3B_2_ and 3B_3_) correspond to the lower, middle, and upper parts of the equilibria curve in Fig 3A (Fig 3B), respectively. The pair of equilibria for *y*^*a*^ and *y*^*b*^ are shown with the same color and linestyle. For example, if *y*^*a*^ is an equilibrium point located on the blue solid-line in Fig 3A_1_, the corresponding equilibrium point *y*^*b*^ is also located on the blue solid-line in Fig 3B_3_. Fig 3 shows that the equilibria of *y*^*a*^ and *y*^*b*^ are not necessarily identical even though the underlying network has symmetric structure. Consequently, different EEG time series can be observed at each region with a suitable initialization.

**Fig 4:**
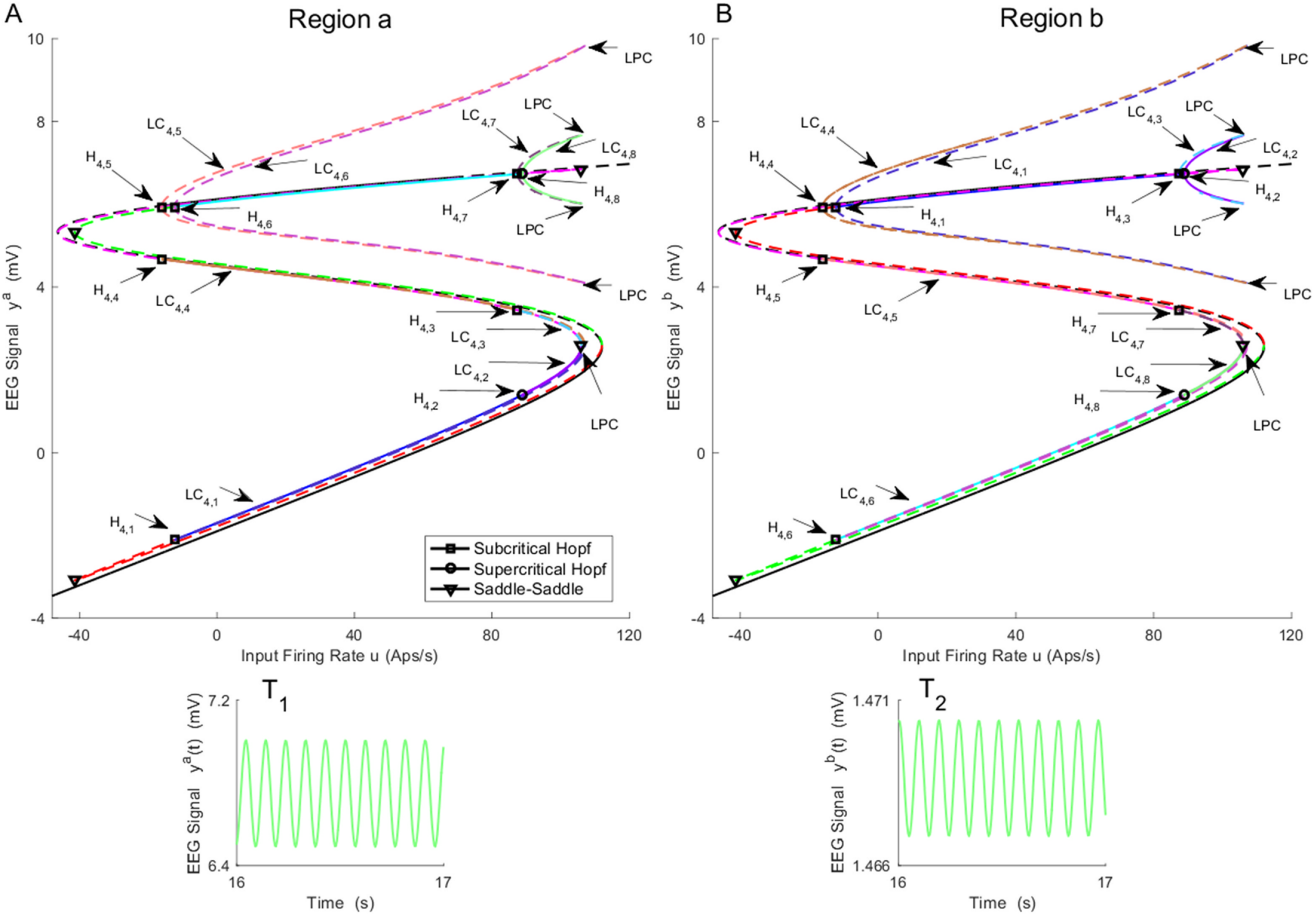
Bifurcation diagrams of A) region *a* and B) region *b* arising from the asymmetric equilibria for case I with coupling gain *K* = 25. The time series in Panels T_1_-T_2_) show the behaviors associated with the stable limit cycle arises from supercritical Hopf bifurcation H_4,8_. The magnified parts around bifurcation points are plotted in Figs 5 and 6. The initial conditions and the corresponding values of the input *u* for all times series are provided in Appendix B.

**Fig 5:**
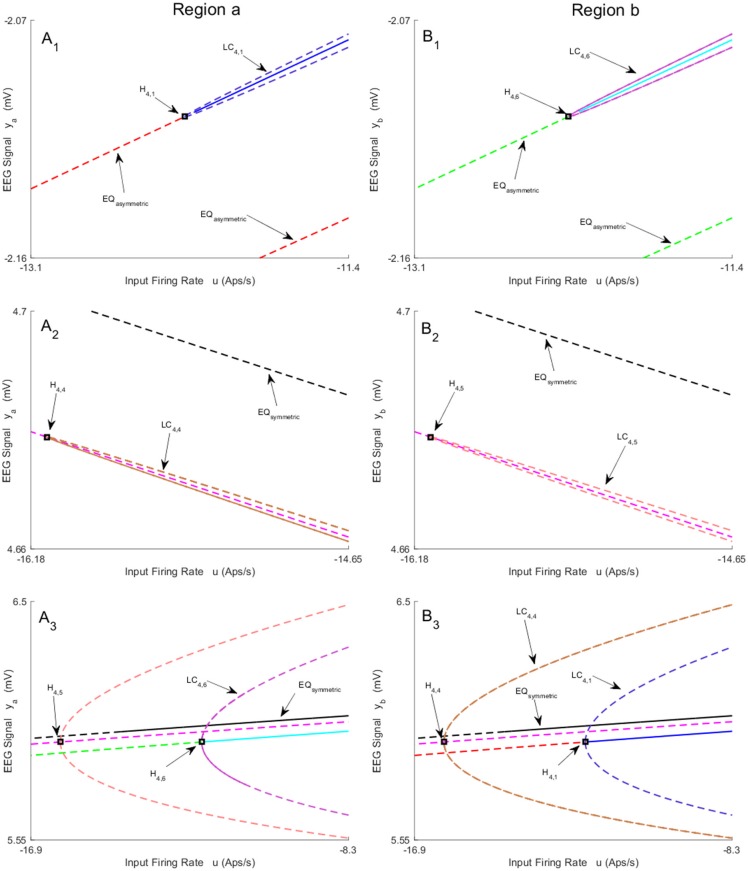
Magnified parts from bifurcation diagrams in Fig 4. A_1_-A_3_) and B_1_-B_3_) show the magnified parts of Panel A) and Panel B). Panels A_1_-A_3_) depict the magnified parts of the bifurcation diagram around H_4,1_, H_4,4_, and H_4,5_ − H_4,6_ respectively. Panels B_1_-B_3_) depict the magnified parts of the bifurcation diagram around H_4,6_, H_4,5_, and H_4,4_ − H_4,1_ respectively. In this figure, EQ_(a)symmetric_ refers to the (a)symmetric equilibria.

**Fig 6:**
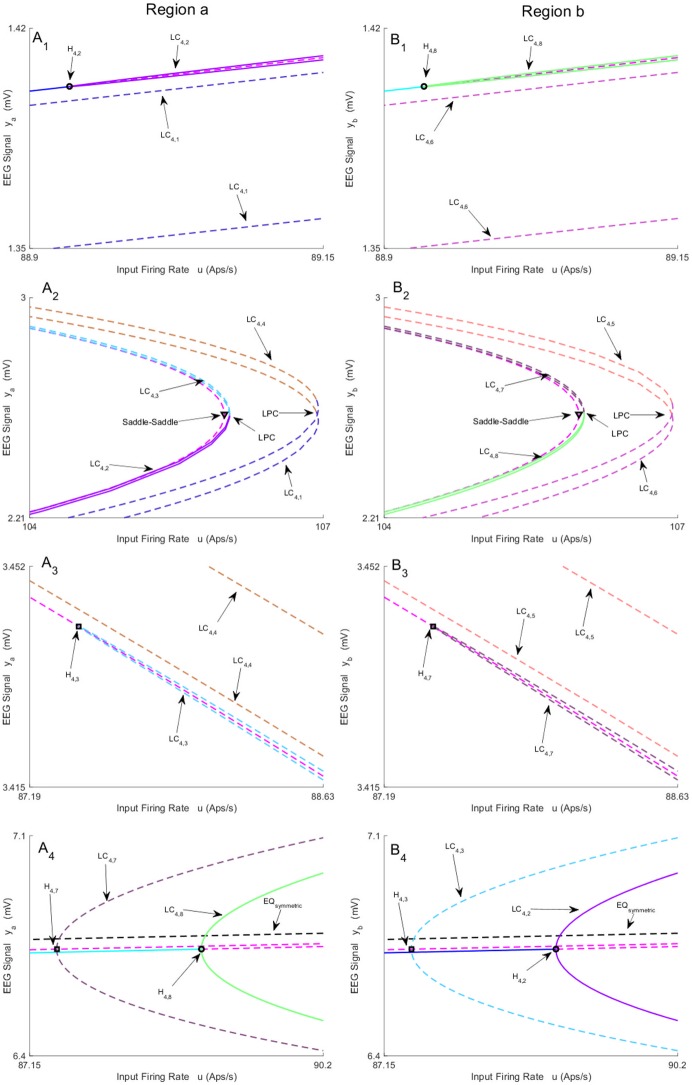
Magnified parts from bifurcation diagrams in Fig 4. A_1_-A_4_) and B_1_-B_4_) show the magnified parts of Panel A) and Panel B) in Fig 4. Panels A_1_-A_4_) depict the magnified parts of the bifurcation diagram around H_4,2_, LPC, H_4,3_ and H_4,7_ − H_4,8_ respectively. Panels B_1_-B_4_) depict the magnified parts of the bifurcation diagram around H_4,8_, LPC, H_4,7_ and H_4,3_ − H_4,2_ respectively. In this figure, EQ_(a)symmetric_ refers to the (a)symmetric equilibria.

All bifurcations found for the asymmetric equilibria are plotted in Figs 4–6 for both regions. The values of *u* for all bifurcation points for asymmetric case are presented in Table 3. Panels A (A_1_ − A_4_) and B (B_1_ − B_4_) show the bifurcation structures for regions *a* and *b*, respectively. Simultaneous bifurcation points and corresponding limit cycles in both panels are color coded. During continuation, we found six subcritical Hopf bifurcations H_4,1_, H_4,3_, H_4,4_, H_4,5_, H_4,6_ and, H_4,7_ that are located in different parts of equilibria curve, and lead to the appearance of six unstable limit cycles (see Figs 5, 6A_3_, 6A_4_, 6B_3_ and 6B_4_). Two limit cycles LC_4,1_, LC_4,4_ (LC_4,5_, LC_4,6_), plotted in same color, collide via a fold bifurcation of limit cycles (or Limit Point of Cycle (LPC)) at *u* = 106 (see Fig 6A_2_ and 6B_2_), which is interesting from a technical perspective since it is a point where a limit cycle is born under other parameter variations.

**Table 3:** The values of input *u* at bifurcation points in Fig 4.

Bifurcation	H_4,1_	H_4,2_	H_4,3_	H_4,4_	H_4,5_	H_4,6_	H_4,7_	H_4,8_
Values of *u*	-12.27	88.93	87.43	-16.10	-16.10	-12.27	87.43	88.93

We also found two supercritical Hopf bifurcations for non-symmetric equilibria that are indicated by H_4,2_ and H_4,8_, and are located in different parts of equilibria curve. The supercritical Hopf bifurcations H_4,8_ and H_4,2_ occur at *u* = 88.93. As a result, two stable limit cycles appear, which generate alpha-like oscillation (10 Hz). The corresponding behavior for LC_4,8_ is show in Fig 4T_1_ and 4T_2_ (the initial conditions and the corresponding values of the input *u* for all times series are provided in Appendix B). However, similar behavior is generated by other stable limit cycle LC_4,2_ with different amplitude. The limit cycles LC_4,8_, LC_4,7_ (LC_4,2_, LC_4,3_), plotted in the same color, collides via LPC at *u* = 106 as shown in Fig 6A_2_ and 6B_2_.

By considering all limit cycles detected from the symmetric and asymmetric branches of equilibria, it is concluded that the network can generate alpha-like activity for the input ranges 87.43 ≤ *u* ≤ 106 and 71.56 ≤ *u* ≤ 313.4, that correspond to the asymmetric and the symmetric cases, respectively. This dynamical regime is vastly more complex than a single region model, which generates alpha activity for 89.83 ≤ *u* ≤ 315.70 from one stable limit cycle [20].

#### 2.1.2 Bifurcation analysis with intermediate coupling (*K* = *50*)

The bifurcation diagram for case I with coupling gain *K* = 50 is qualitatively similar to the case *K* = 25 in terms of types of limit cycles and shapes of equilibria branches. The differences between *K* = 25 and 50 are the points at which the bifurcations occur and the amplitudes of oscillations. Similar to the case *K* = 25, two orbits, resulting from a saddle-node homoclinic bifurcation coexist for 110.5 ≤ *u* ≤ 114.3 and 106.2 ≤ *u* ≤ 134.4. Two stable limit cycles emerge from supercritical Hopf bifurcations at *u* = 53.24 and *u* = 97.2 and vanish at *u* = 310.5 and *u* = 280.7, respectively. A similar situation to the case *K* = 25 is observed for the limit cycle corresponding to the second branch of equilibria. Due to these similarities, the bifurcation diagrams are not presented.

#### 2.1.3 Bifurcation analysis with strong coupling (*K* = *100* and *150*)

**Fig 7:**
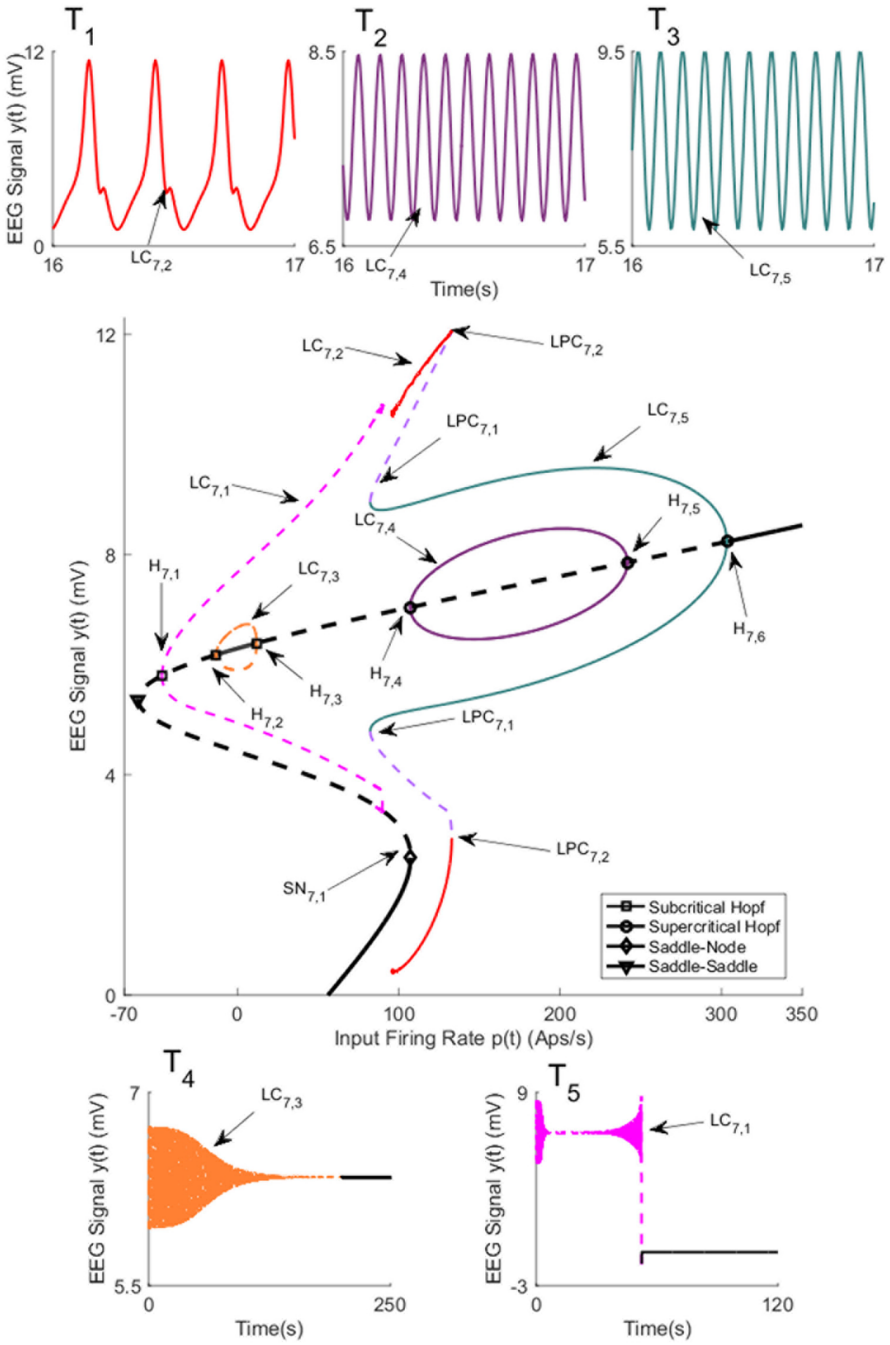
Bifurcation diagrams arising from the first branch of equilibria in case I with coupling gain *K* = 100. The time series in Panels T_1_-T_5_) show the simulated EEG for each bifurcation with the same color. The solid black lines show stable fixed points, the solid colored lines show stable oscillatory behavior and the dashed lines show unstable fixed points and unstable oscillations. The initial conditions and the corresponding values of the input *u* for all times series are provided in Appendix B.

The symmetric solutions to the equilibria were computed and are plotted in Fig 7 for case I with strong inter-region coupling gain (*K* = 100). The values of *u* for all bifurcation points for symmetric case are presented in Table 4. Fig 7 shows two stable limit cycles LC_7,4_ and LC_7,5_ that originate from supercritical Hopf bifurcations H_7,4_(H_7,5_) and H_7,6_ respectively. The first limit cycle LC_7,4_ occurs for the input range 107.1 ≤ *u* ≤ 241.7. The limit cycle LC_7,5_ arises as a result of LPC with an unstable limit cycle at the indicated point LPC_7,1_, and terminates at *u* = 303.3. The frequency of oscillations from both LC_7,4_ and LC_7,5_ is approximately 10 Hz, corresponding to alpha-like activity as shown in Fig 7T_2_ and 7T_3_ (the initial conditions and the corresponding values of the input *u* for all times series are provided in Appendix B). Contrary to the network with weak coupling (*K* = 25), there is only one limit cycle LC_7,2_ that generates spike-wave-like discharges. The limit cycle LC_7,2_ arises from a saddle-node homoclinic bifurcation, denoted by *SN*_7,1_, and collides with an unstable limit cycle through LPC at the point indicated by LPC_7,2_. The frequency and amplitude of spikes for this case are approximately the same as with weak coupling.

**Table 4:** The values of input *u* at bifurcation points in Fig 7.

Bifurcation	H_7,1_	H_7,2_	H_7,3_	H_7,4_	H_7,5_	H_7,6_	SN_7,1_
Values of *u*	-46.74	-13.28	11.92	107.1	241.7	303.3	107.4

There are two subcritical Hopf bifurcations H_7,1_ and H_7,2_ that generate the unstable limit cycles LC_7,1_ and LC_7,3_ respectively. The limit cycle LC_7,1_ emerges when the input *u* = −46.74. We couldn’t identify how LC_7,1_ ends by increasing *u* as CL-MATCONT package was not able to proceed the continuation process further. The limit cycle LC_7,3_ begins at *u* = −13.28 and ends at *u* = 11.92 through Hopf bifurcation H_7,3_. The EEG time series in the bottom part of Fig 7 shows decaying oscillations that settle down to constant values corresponding to stable equilibria.

**Fig 8:**
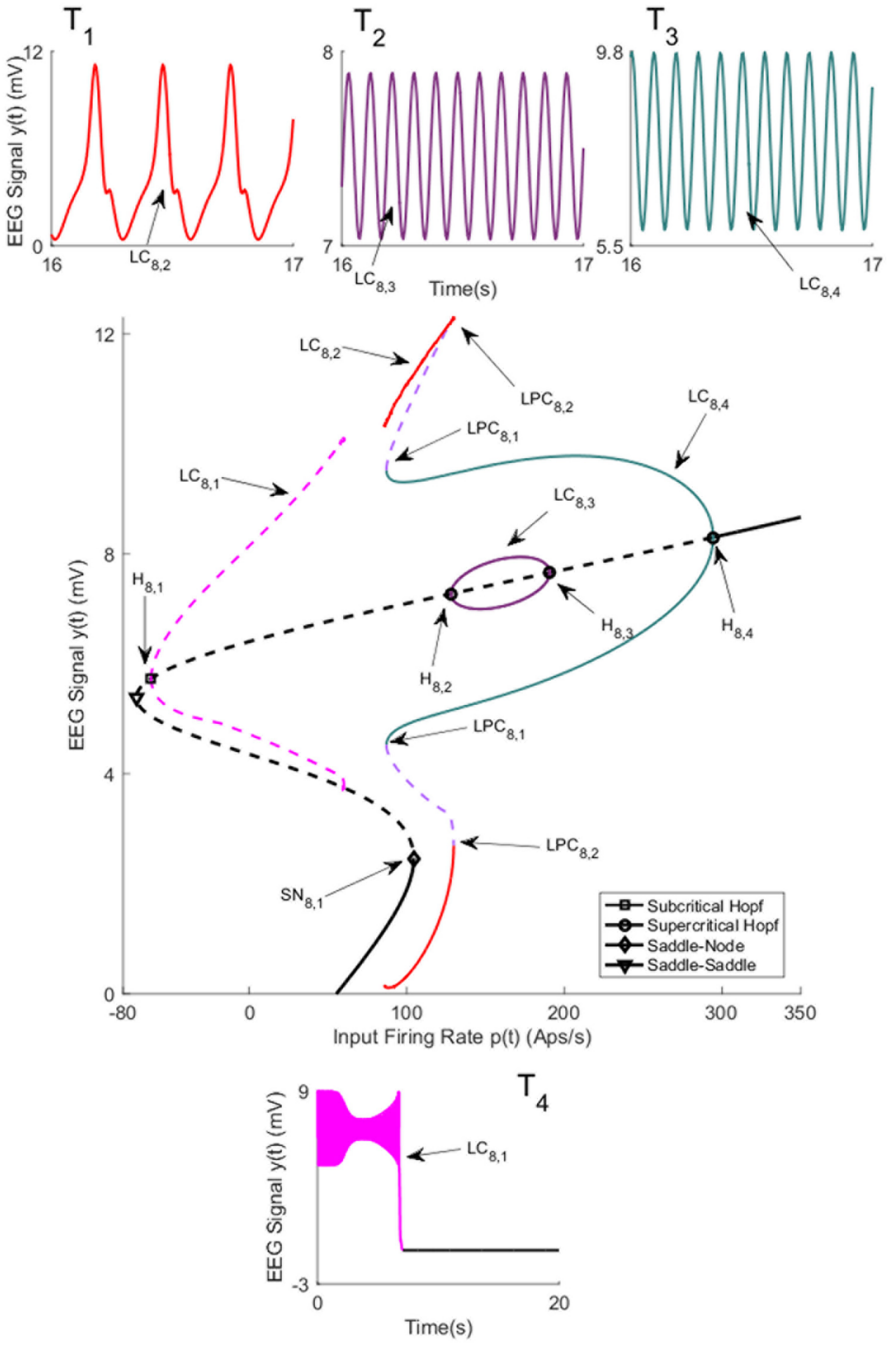
Bifurcation diagrams arising from the first branch of equilibria in case I with coupling gain *K* = 150. The time series in Panels T_1_-T_4_) show the simulated EEG for each bifurcation with the same color. The solid black lines show stable fixed points, the solid colored lines show stable oscillatory behavior and the dashed lines show unstable fixed points and unstable oscillations. The initial conditions and the corresponding values of the input *u* for all times series are provided in Appendix B.

For coupling gain *K* = 150, the network has two branches of equilibria that correspond to the symmetric and asymmetric parts. The bifurcation diagram for the symmetric equilibria for *K* = 150 is plotted in Fig 8. The values of *u* for all bifurcation points for symmetric case are presented in Table 5. The initial conditions and the corresponding values of the input *u* for all times series are also provided in Appendix B. The diagram has a notable exception of the disappearance of the small unstable limit cycle LC_7,3_ which results from a subcritical Hopf bifurcation for *K* = 100. The reason is that, for the corresponding range of *u*, the Jacobian matrix for the system 4 has no complex eigenvalue with zero real part. There are also differences in the levels of the input at which other types of bifurcations arise. For the asymmetric case of equilibria, there are two unstable limit cycles, arising from subcritical Hopf bifurcations, that do not lead to any interesting behavior and are not discussed further.

**Table 5:** The values of input *u* at bifurcation points in Fig 8.

Bifurcation	H_8,1_	H_8,2_	H_8,3_	H_8,4_	SN_8,1_
Values of *u*	-62.21	128	190.7	294.4	104.4

### 2.2 Case II: Bifurcation analysis of two coupled neural mass models with a single input

In this section, the bifurcation analyses of the neural mass model network are presented where the input is applied only to region *a* (see Fig 1). Similar to Section 2.1, the first step of the bifurcation analysis is finding the equilibria of the overall system. We follow the procedure in Section 1.3, setting *u*^*b*^ to zero. Additional notes on calculating the equilibria for this case are provided in Appendix C.

#### 2.2.1 Bifurcation analysis with coupling gain *K* = *50*

**Fig 9:**
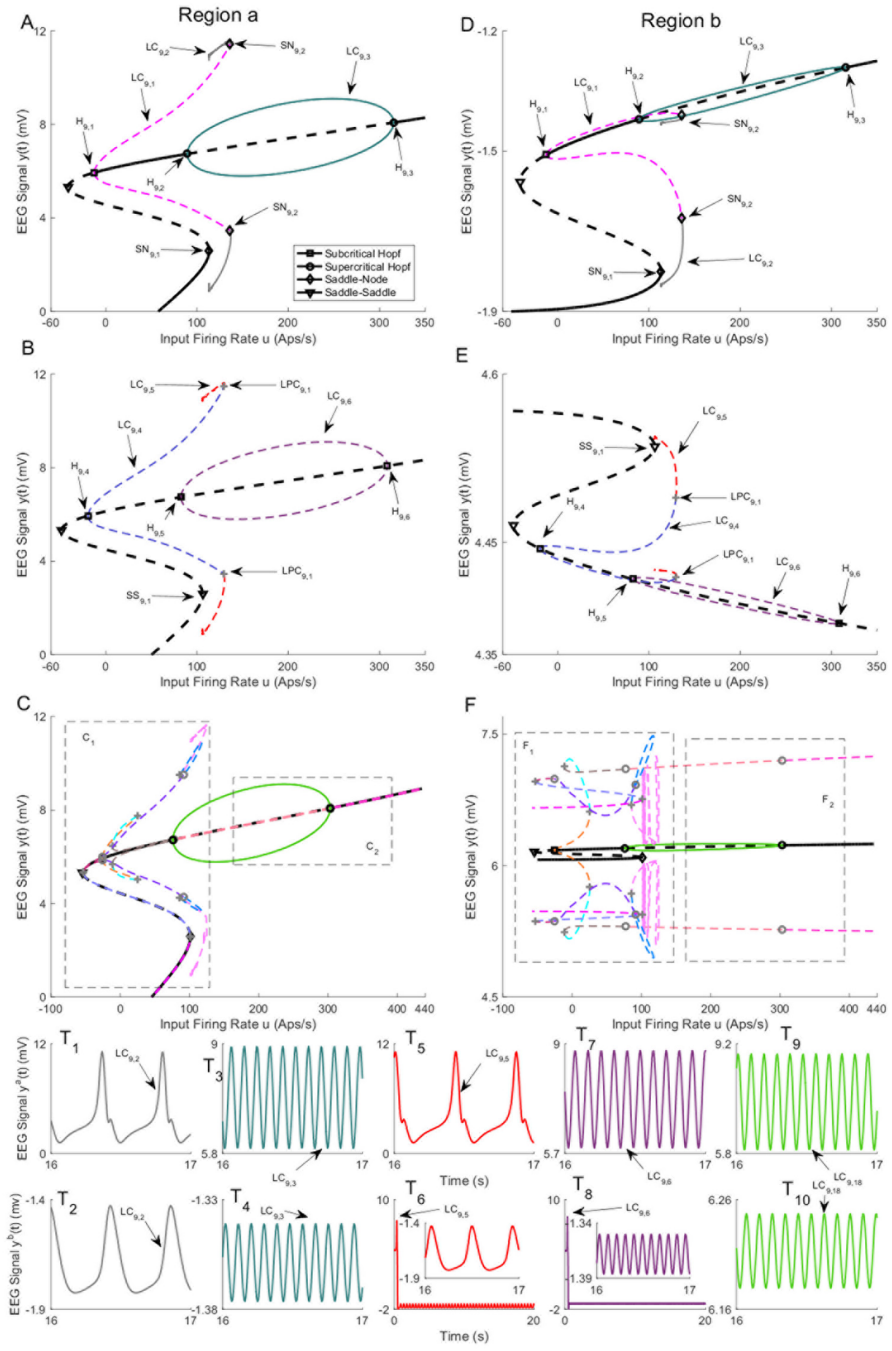
Equilibria and bifurcation diagrams for case II with coupling gain *K* = 50. A), B), and C) are the first, second, and third branches of equilibria for region *a*. D), E), and F) are the first, second, and third branches of equilibria for region *b*. Panels T_1_-T_10_) show the EEG time series corresponding to the each part in the bifurcation diagrams. The solid black lines show stable fixed points, the solid colored lines show stable oscillatory behavior and the dashed lines show unstable fixed points and unstable oscillations. The initial conditions and the corresponding values of the input *u* for all times series are provided in Appendix B.

**Fig 10:**
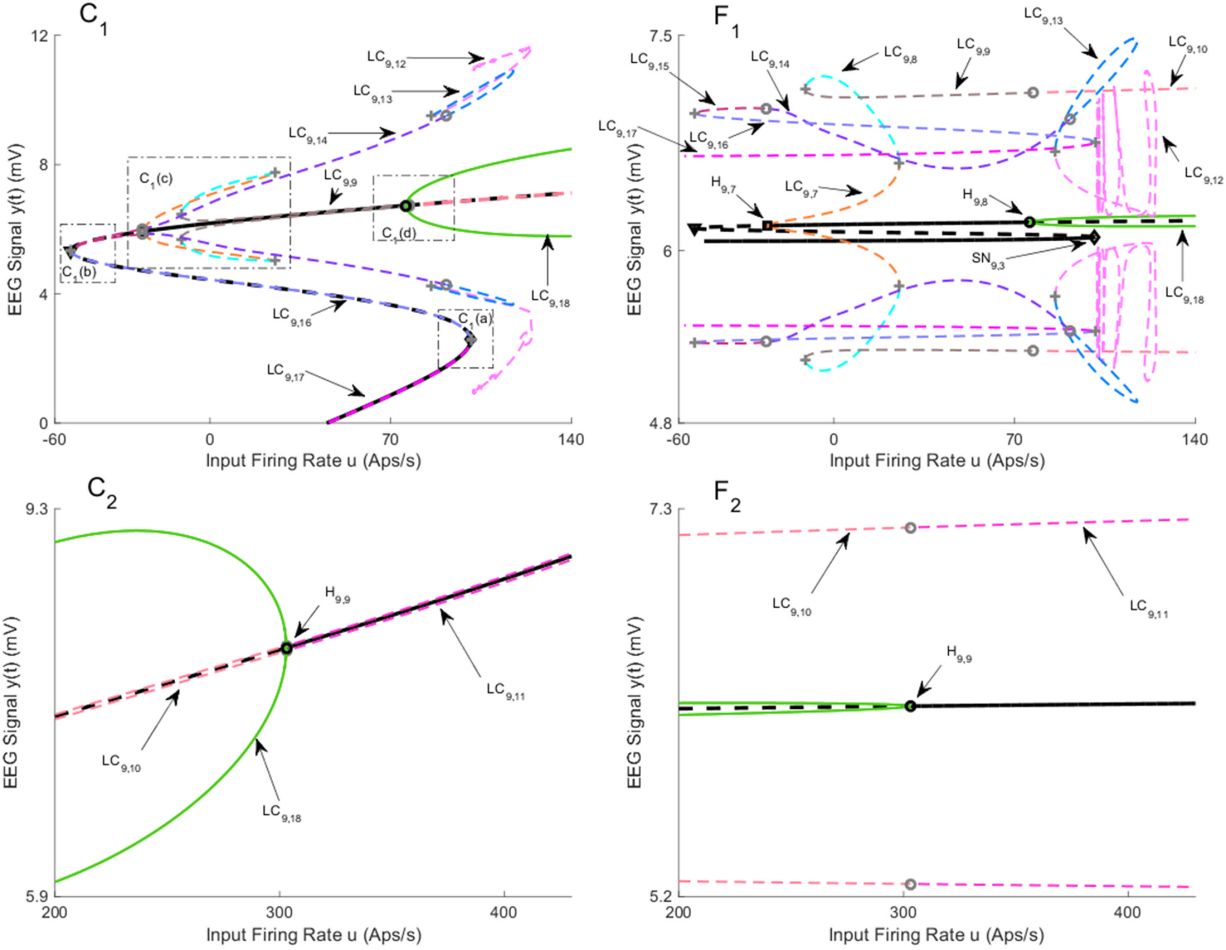
Magnified parts from the bifurcation diagram of the third branch of equilibria in Fig 9C and 9F. Panels C_1_ and C_2_ show the magnified parts of bifurcation diagram in Fig 9C that are indicated by C_1_ and C_2_, respectively. Panels F_1_ and F_2_ show the magnified parts pf bifurcation diagram in Fig 9F that are indicated by F_1_ and F_2_, respectively.

**Fig 11:**
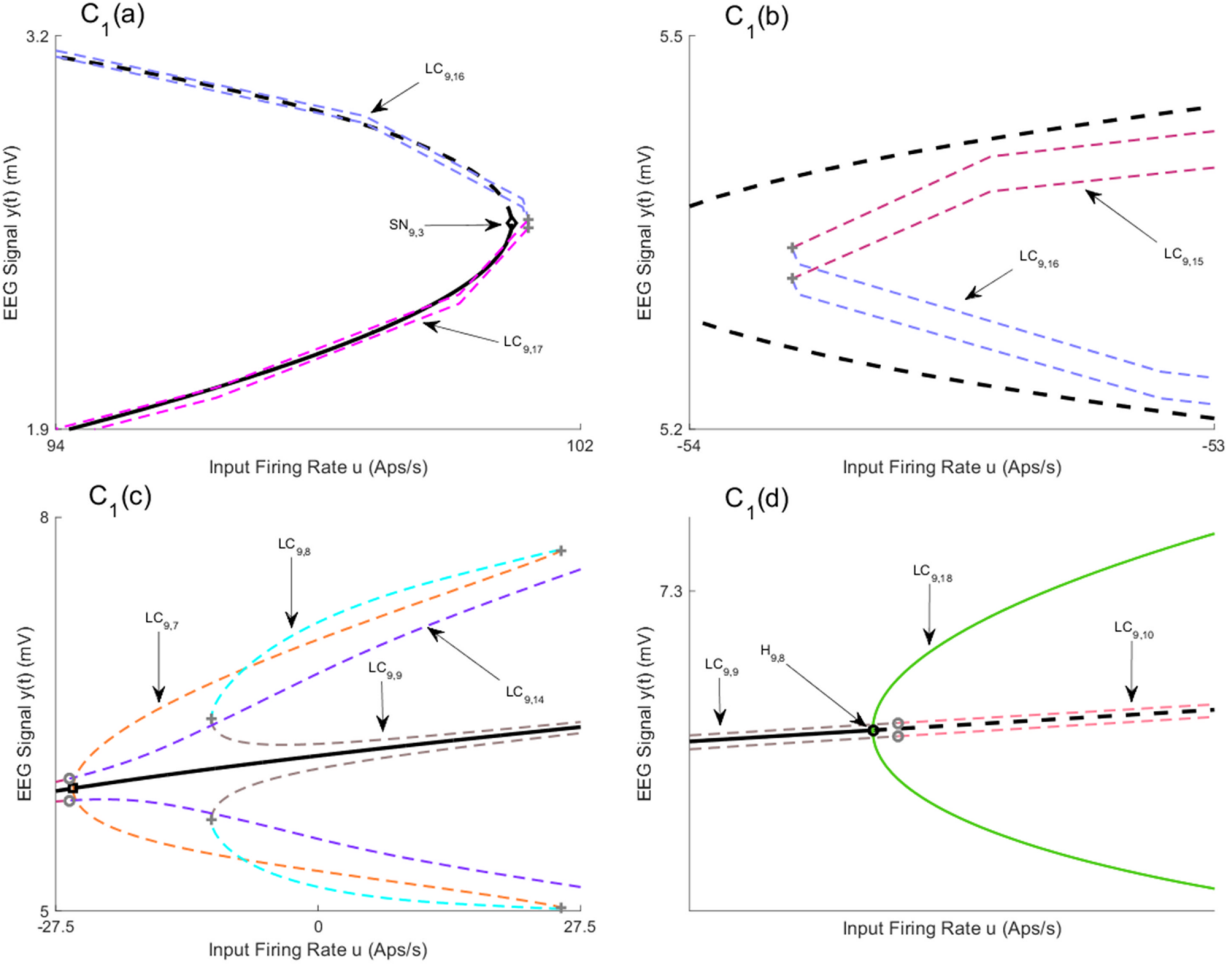
Magnified parts from the bifurcation diagram of the third branch of equilibria in Fig 10C_1_. Panels C_1_(a), C_1_(b), C_1_(c) and C_1_(d) show the magnified parts of bifurcation diagram in Fig 10C_1_ that are indicated by C_1_(a) and C_1_(b), C_1_(c) and C_1_(d), respectively.

Fig 9 depicts three branches of equilibria and bifurcation diagrams for region *a* and region *b*. In the sequel, we refer to the equilibria in Fig 9A, 9D, 9B, 9E, 9C and 9F by the first, second, third branch of equilibria, respectively. Since the bifurcation diagrams in Fig 9C and 9F are complicated, their magnified parts are depicted in Figs 10 and 11. The values of *u* for all bifurcation points are presented in Table 6. These figures illustrate that the equilibria of region *a* are very similar between all three branches. However, the equilibria for region *b* have a more complex structure (see Appendix C for further explanation).

**Table 6:** The values of input *u* at bifurcation points in Figs 9, 10 and 11.

Bifurcation	H_9,1_	H_9,2_	H_9,3_	SN_9,1_	SN_9,2_
Values of *u*	-12.5	89.43	315.3	113.3	136.1
Bifurcation	H_9,4_	H_9,5_	H_9,6_	SS_9,1_	
Values of *u*	-19.34	82.55	308.7	106.1	
Bifurcation	H_9,7_	H_9,8_	H_9,9_	SN_9,3_	
Values of *u*	-25.7	75.98	302.9	100.9	

There are two stable limit cycles LC_9,3_ (Fig 9A and 9D) and LC_9,18_ (Figs 9C, 9F and 10C_1_, 10C_2_, 10F_1_ and 10F_2_) on the first and the third branches of equilibria that exist between supercritical Hopf bifurcation points (H_9,2_, H_9,3_) and (H_9,8_, H_9,9_), respectively. These limit cycles exist for input values roughly between *u* = 83 and *u* = 300 (see Table 6 for the exact value of *u*). Similar to Case I, supercritical Hopf bifurcations lead to stable limit cycles which generates stable oscillations, depicted in Fig 9T_3_, 9T_4_, 9T_9_ and 9T_10_, that resemble alpha activity (the initial conditions and the corresponding values of the input *u* for all times series are provided in Appendix B). These two limit cycles generate different types of alpha activity with the same frequency at distinctly different amplitude ranges in region *b*. In order to study the behavior of the network near the stable limit cycles, we simulated the EEG signals with initial conditions close to the cycles and plotted the corresponding time series shown in the lower part of Fig 9. The time series associated with the stable limit cycles on the first and third branches verify that the limit cycles are stable.

We found several subcritical Hopf bifurcations on all branches of equilibria. The one for the first branch H_9,1_ results in the unstable limit cycle LC_9,1_. This limit cycle collides with the limit cycle LC_9,2_ via a saddle-node bifurcation at the point SN_9,2_. The limit cycle LC_9,2_ originates from a saddle-node homoclinic bifurcation at the point SN_9,1_. The stable limit cycle LC_9,2_ produces spike-wave-like signals with a frequency of approximately 3 Hz, which is observed in region *a* (Fig 9T_1_). However, the spike-wave-like signal does not appear in region *b* as shown in Fig 9T_2_. Instead, region *b* shows an EEG signal similar to delta-wave activity (Fig 9T_2_). The occurrence of delta wave activity is interesting considering the strong links between epileptic seizures and sleep [1].

The unstable limit cycle LC_9,4_ in Fig 9B and 9E originates from subcritical Hopf bifurcation H_9,4_, and appears to collide with the limit cycle LC_9,5_ at *u* = 129.4. At this point indicated by LPC_9,1_, LPC was detected. The limit cycle LC_9,5_ originates from a homoclinic bifurcation of a saddle-saddle (denoted by SS_9,1_ in Fig 9B and 9E) which was originally proposed by Shilnikov (see http://www.scholarpedia.org/article/Shilnikov_saddle-node_bifurcation). We observed that the trajectories initialized near the limit cycle LC_9,4_ converge to the equilibria on the first branch. Furthermore, the trajectories that initialized near the limit cycle LC_9,5_, depicted in Fig 9T_5_ and 9T_6_, converge to the LC_9,2_ on the first branch of equilibria. There are two subcritical Hopf bifurcations H_9,5_ and H_9,6_ for the second branch of equilibria that lead to the existence of the unstable limit cycle LC_9,6_. By initializing the system near to this limit cycle, the trajectories converge to the limit cycle LC_9,3_ as shown in Fig 9T_7_ and 9T_8_. Therefore, the analysis indicates that this second branch does not contribute to specific behaviors.

Fig 9C and 9F shows the bifurcation diagram for the third branch of equilibria that are magnified and labeled in Figs 10 and 11. In contrast to the bifurcation diagram for the first branch of equilibria, the unstable limit cycle LC_9,7_ (Figs 10C_1_, 10F_1_ and 11C_1_(a)), which arises from subcritical Hopf bifurcation H_9,7_, does not collide with the unstable limit cycle LC_9,12_ (Fig 10C_1_ and 10F_1_) resulted from a saddle-node homoclinic bifurcation SN_9,3_ (Figs 10C_1_, 10F_1_ and 11C_1_(a)). During continuation of the third limit cycle LC_9,7_, we detected LPC, which is plotted by a gray plus sign. At this point, the limit cycle LC_9,7_ collides with the limit cycle LC_9,8_ (see Fig 10C_1_, 10F_1_ and 10C_1_(c)). By proceeding the continuation, we detected another LPC point. We labeled the limit cycle after this point as LC_9,9_. We also noticed that the toolbox detected Neimark-Sacker bifurcation of limit cycles. Neimark-Sacker bifurcation of cycles is a co-dimension 1 bifurcation corresponds to the case when the multipliers are complex and simple and lie on the unit circle. (see [31] for more details). denoted by gray red circles, at two points; (i) the intersection of limit cycles LC_9,9_ and LC_9,10_ (Figs 10F_1_ and 11c_1_(d)) (ii) the intersection of limit cycles LC_9,10_ and LC_9,11_ (Fig 10C_2_ and 10F_2_). To study the simulated EEG corresponding to these limit cycles, the initial value was chosen near each limit cycle. We observed that trajectories converge to either the equilibria on the third branch or the equilibrium point on the first branch.

Near the saddle node point SN_9,3_ (Figs 10C_1_, 10F_1_ and 11C_1_(a)) on the third branch, we noticed that there exists a saddle-node homoclinic bifurcation, which results in an appearance of the limit cycle LC_9,12_ (Fig 10C_1_ and 10F_1_). By initializing the system close to this limit cycle, we observed that it produces unstable spikes in region *a* and an oscillation in region *b*. These spike-wave discharges have a frequency similar to that observed during seizures in clinical EEG recordings, until the activity of each region settles to the equilibrium point. During the continuation of this cycle, three LPCs and one Neimark-Sacker bifurcation of cycles are detected (see Figs 10C_1_, 10F_1_, 11c_1_(a), 11c_1_(b) and 11c_1_(c)). By selecting an initial condition near each limit cycle and simulating the EEG, we observed the solutions converge to the equilibria on either the first branch or the third branch.

#### 2.2.2 Bifurcation analysis with coupling gain *K* = *250*

**Fig 12:**
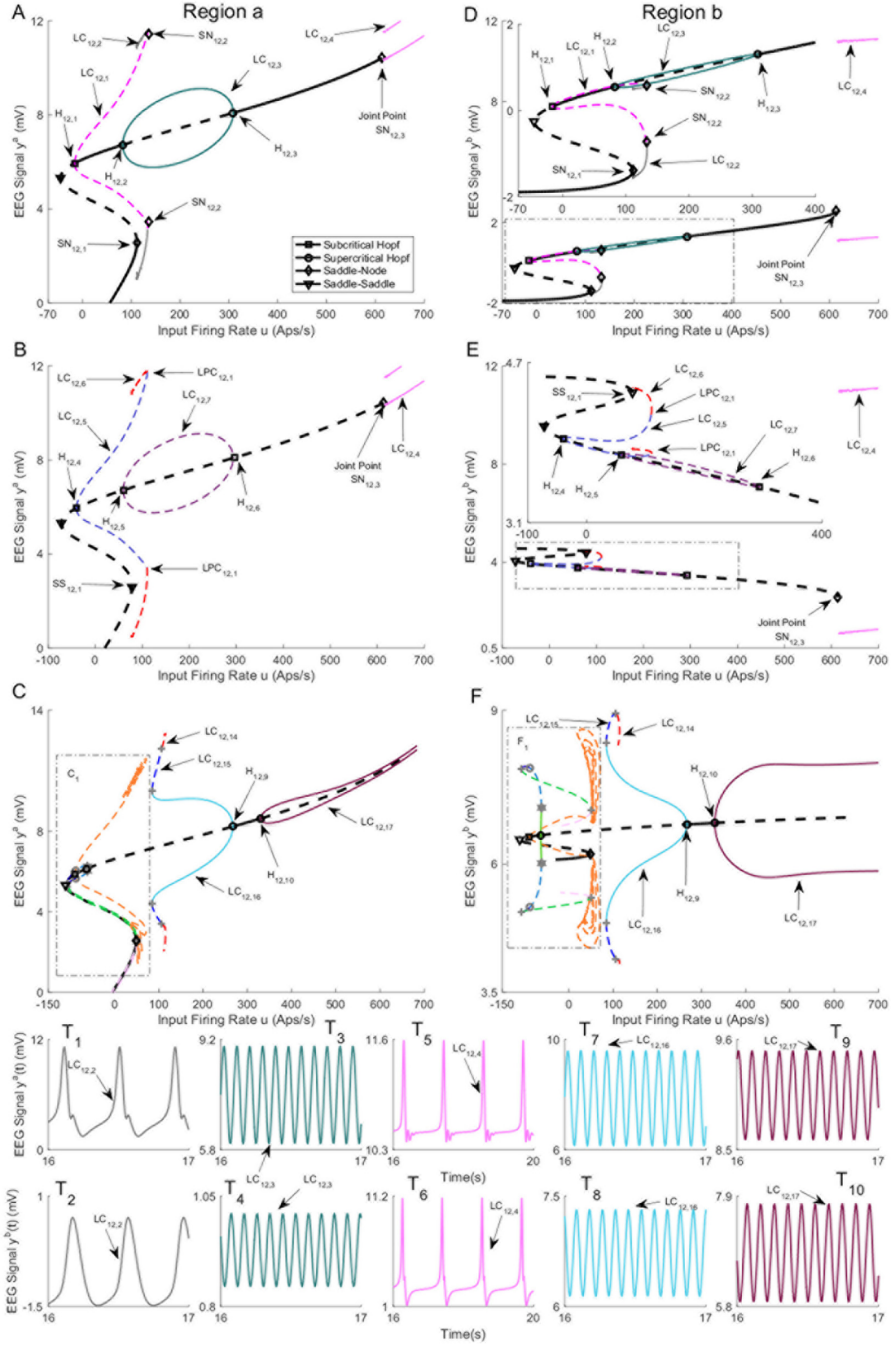
Bifurcation diagrams for case II with coupling gain *K* = 250. A) and B) are the bifurcation diagrams for the first branch of equilibria for region *a*, and C) is the bifurcation diagram for the second branch of equilibria for region *a*. D) and E) are the bifurcation diagrams for the first branch of equilibria for region *b*, and F) is the bifurcation diagram for the second branch of equilibria for region *b*. Panels T_1_-T_10_) show the EEG time series corresponding to the each part in the bifurcation diagrams. The solid black lines show stable fixed points, the solid colored lines show stable oscillatory behavior and the dashed lines show unstable fixed points and unstable oscillations. The initial conditions and the corresponding values of the input *u* for all times series are provided in Appendix B.

By increasing the coupling gain to *K* = 250, two branches of equilibria for region *b* join up, which results in the appearance of a saddle node in the joint point (refer to Appendix C). As a consequence, the new saddle-node homoclinic bifurcation starts that leads to new behavior in the network, such as observing spikes in only one region or in both regions for all inputs larger than the value at which the saddle node arises. Fig 12 shows all equilibria branches and bifurcations that are detected in this case. In order to present this case, we split the first branch of equilibria from the saddle point and present them in different sub-figures (Fig 12A and 12B for equilibria of region *a*, and Fig 12D and 12E for equilibria of region *b*). The values of *u* for all bifurcation points for symmetric case are presented in Table 7.

**Table 7:** The values of input *u* at bifurcation points in Figs 12 and 13.

Bifurcation	H_12,1_	H_12,2_	H_12,3_	SN_12,1_	SN_12,2_
Values of *u*	-15.99	82.59	308.7	111.6	133.3
Bifurcation	H_12,4_	H_12,5_	H_12,6_	SS_12,1_	SN_12,3_
Values of *u*	-39.86	59.82	293.2	77.62	613.7
Bifurcation	H_12,7_	H_12,8_	H_12,9_	H_12,10_	SN_12,4_
Values of *u*	-89.2	-63.13	267.2	330.1	48.49

Fig 12A, 12B, 12D and 12E show the bifurcation diagram from the first branch of equilibria. There are six Hopf bifurcations detected on this branch (H_12,1_-H_12,6_) and only two of them (H_12,2_,H_12,3_,) are supercritical. Fig 12A and 12D illustrate three limit cycles LC_12,1_−LC_12,3_ that arise from the bottom part of this branch. Similar to the case of *K* = 50, the unstable limit cycle LC_12,1_ collides with the limit cycle LC_12,2_ that appears from the saddle-node homoclinic bifurcation. The time series associated with the limit cycle LC_12,2_, depicted in Fig 12T_1_ and 12T_2_, verify that the limit cycle causes region *a* to produce spikes while region *b* generates delta activity (the initial conditions and the corresponding values of the input *u* for all times series are provided in Appendix B). Furthermore, the stable limit cycle LC_12,3_, results from supercritical Hopf bifurcation, provokes alpha activity in both regions as shown in Fig 12T_3_ and 12T_4_. The bifurcation analysis of the top part of the first equilibria branch, shown in Fig 12B and 12E, is similar to the second branch of equilibria of the previous case; the trajectories of the network initialized near the limit cycles LC_12,5_ − LC_12,7_ are either attracted by the stable limit cycles or attracted by the stable equilibria on the bottom part of the first equilibrium curves.

From a topological point of view, the differences between the two cases with coupling gains *K* = 50 and *K* = 250 emerge from limit cycles that arise from the third branch of equilibria and the appearance of a limit cycle from the saddle-node homoclinic bifurcation on the first branch. The bifurcation analysis shows that the limit cycle LC_12,4_ starts near the saddle-node homoclinic bifurcation on the first branch of equilibria, denoted by SN_12,3_ in Fig 12A, 12B, 12D and 12E for *u* = 613.7, and it exists for all values of input larger than *u* = 613.7, which means that the underlying network can generate spikes in both regions for large values of *u* in contrast to all previous cases in which spikes disappear for large values of *u*. The time series associated with the limit cycle LC_12,4_, shown in Fig 12T_5_ and 12T_6_, verify that this limit cycle generates spikes in both regions.

**Fig 13:**
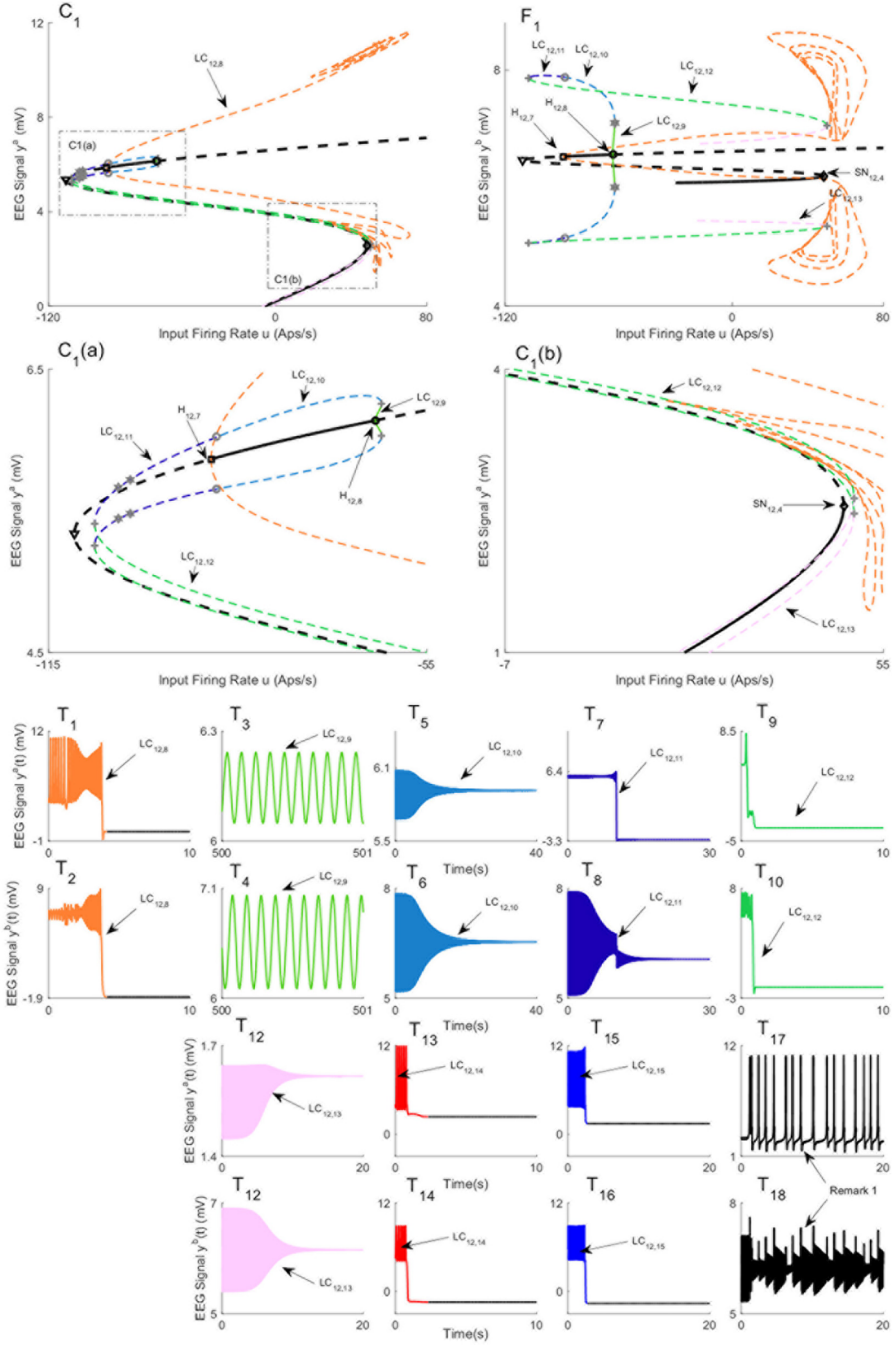
Magnified parts from the bifurcation diagram of the third branch of equilibria in Fig 12C and 12F. The panels C_1_) and F_1_) show the magnified parts of bifurcation diagram in Fig 12C and 12F that are indicated by C_1_ and F_1_, respectively. Panels C_1_(a)) and C_1_(b)) show the magnified parts of Panel C_1_). Panels T_1_-T_16_)) show the EEG time series corresponding to the each part in the bifurcation diagrams. The panels T_17_) and T_18_) show the EEG time series for the case in Remark 1. The initial conditions and the corresponding values of the input *u* for all times series are provided in Appendix B.

All limit cycles that emerge from the second branch of equilibria are depicted in Figs 12C, 12F and 13. There are four Hopf bifurcations (H_12,7_-H_12,10_) among which three are supercritical (H_12,8_-H_12,10_). All limit cycles LC_12,9_, LC_12,16_, LC_12,17_, originated from supercritical Hopf bifurcations, generate alpha activity with frequency 11 Hz as depicted in Fig 13T_3_ and 13T_4_ (the initial conditions and the corresponding values of the input *u* for all times series are provided in Appendix B), and Fig 12T_7_–12T_10_. The stable limit cycle LC_12,16_ starts at *u* = 267.2 from supercritical Hopf bifurcation and collides with LC_12,15_ through LPC bifurcation at *u* = 84.73. We also observed that the limit cycle LC_12,15_ collide with the limit cycle LC_12,14_ via LPC at *u* = 107.2. We were not able to proceed the continuation further to check the origin of the limit cycle LC_12,14_. The time responses in Fig 13T_13_–13T_16_ shows that the trajectories of the network near these limit cycles converge to the equilibria on the first branch. The stable limit cycle LC_12,17_ starts at *u* = 330.1 and exists for all values of input larger that *u* = 330.1. As a consequence, the network can also generate alpha activity in both regions for large values of *u*; however, in all previous cases, alpha activity was only observed for values of *u* in finite intervals.

The stable limit cycle LC_12,9_ (see Fig 13) arises from supercritical Hopf bifurcation H_12,6_. However, it collides with the limit cycle LC_12,10_ via LPC as shown in Fig 13F_1_ and 13C_1_(a). By looking at these figures, it is possible to see how different bifurcations lead to different limit cycles. The times series associated with these limit cycles are shown in Fig 13T_1_, 13T_2_ and 13T_5_–13T_12_. We mention that Neimark-Sacker bifurcations of cycles (indicated by gray circle in Fig 13) and period-doubling bifurcations (indicated by gray hexagram in Fig 13) are detected during the continuation.

**Remark 1** We initialized the model to the right from the saddle node SN_12,4_ in order to check the existence of a limit cycle. It seems that there exists a limit cycle which generates the output depicted in Fig 13T_17_ and 13T_18_. We couldn’t do the continuation from this point due to software limitations.

### 2.3 Case III: Bifurcation analysis of two coupled neural mass models with a single input and feed-forward structure

In this section, we present the bifurcation analysis of case III, which is graphically depicted in Fig 1. Similar to previous cases, we start by finding equilibria of the network by solving (7) with *u*^*b*^ and *K*^*ba*^ set to zero. We observe that equilibrium curves for region *b* are qualitatively similar to Case II. However, the equilibrium curves for region *a* are slightly different. Hence, we analyze the bifurcation diagram for the network with interconnection gains *K* = 50 and 250, and explain the important differences.

**Fig 14:**
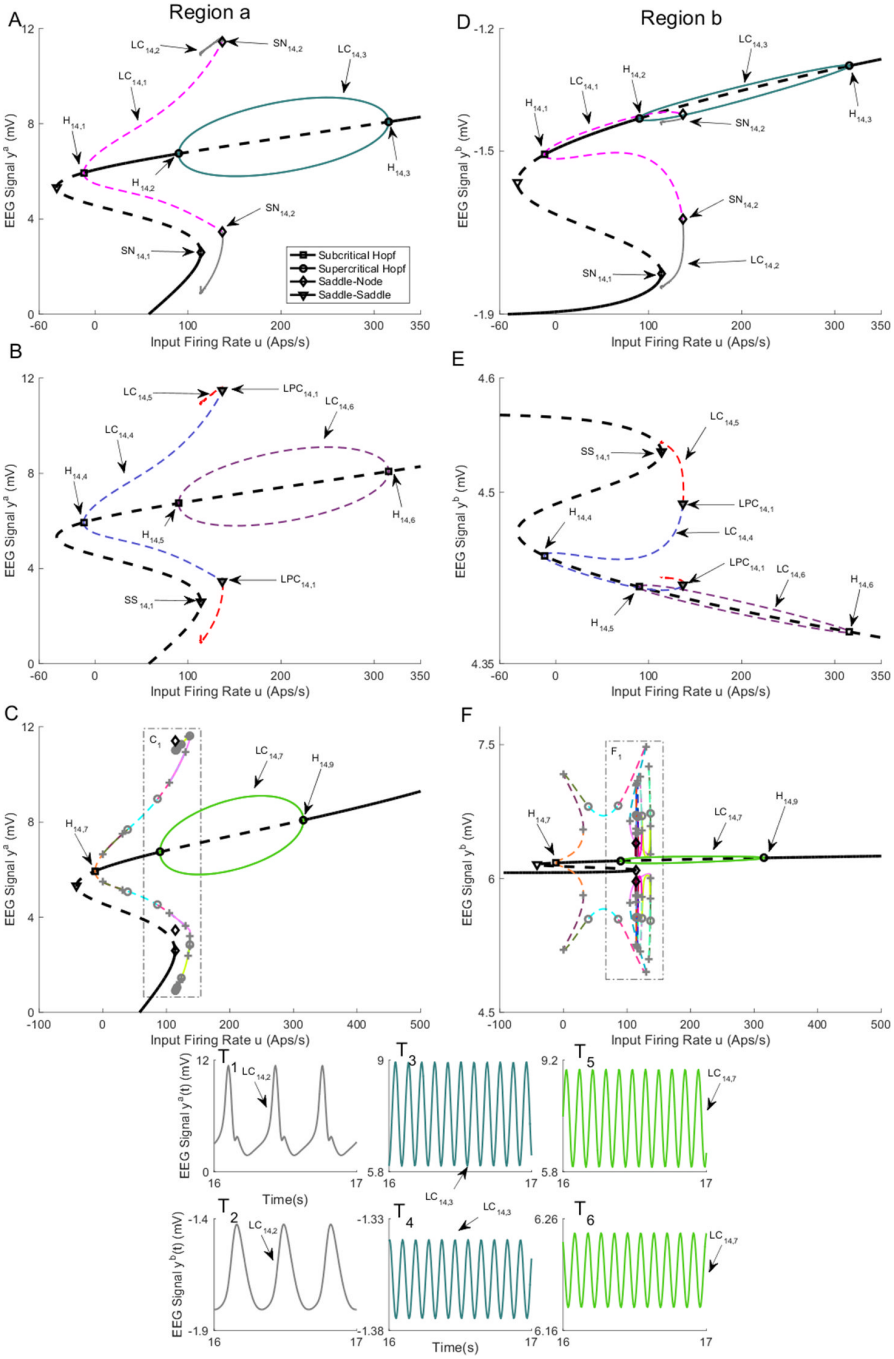
Bifurcation diagrams for case III with coupling gain *K* = 50. A), B), and C) are the first, second, and third branches of equilibria for region *a*. D), E), and F) are the first, second, and third branches of equilibria for region *b*. Panels T_1_-T_6_) show the EEG time series corresponding to the each part in the bifurcation diagrams. The solid black lines show stable fixed points, the solid colored lines show stable oscillatory behavior and the dashed lines show unstable fixed points and unstable oscillations. The initial conditions and the corresponding values of the input *u* for all times series are provided in Appendix B.

**Fig 15:**
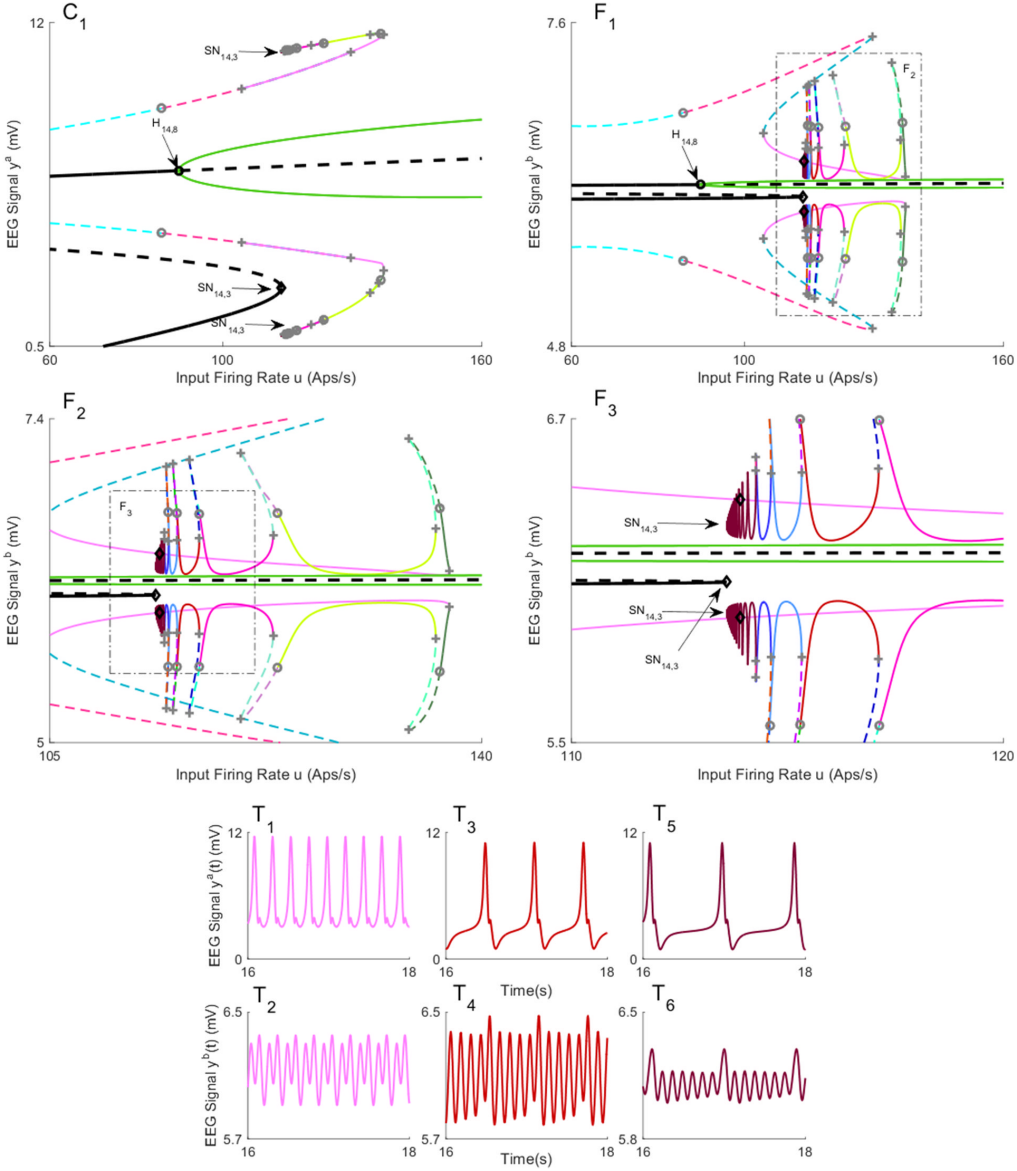
Magnified parts from the bifurcation diagram of the third branch of equilibria in A) Fig 14C and 14B) B) Fig 14F. Panels C_1_) and F_1_) show the magnified parts of bifurcation diagram in Fig 14C and 14F that are indicated by C_1_ and F_1_, respectively. Panels F_2_) show the magnified parts of Panel F_1_). Panel F_3_) show the magnified parts of Panel F_2_). Panels T_1_-T_6_)) show the EEG time series corresponding to the each part in the bifurcation diagrams. The initial conditions and the corresponding values of the input *u* for all times series are provided in Appendix B.

The bifurcation diagrams of the network with *K* = 50 are presented in Figs 14 and 15. The values of *u* for all bifurcation points are presented in Table 8. It can be seen that the bifurcation diagram of the first and second branches of equilibria are qualitatively similar to the case in Section 2.2.1. For the third branch of equilibria, there is a stable limit cycle LC_14,7_ (Fig 14C and 14F) that, similar to the previous cases, produces the alpha activity that is shown in Fig 14T_5_ and 14T_6_ (the initial conditions and the corresponding values of the input *u* for all times series are provided in Appendix B). There is an unstable limit cycle that emerges for *u* = −12.15 from supercritical bifurcation H_14,7_. This limit cycle collides with other limit cycles through LPC as can be seen in Fig 14C and 14F. By continuing along the curve, we detected several LPC points (indicated by gray plus sign), Neimark-Sacker bifurcations of limit cycles (indicated by gray circle). Since there are many of them and consequently many limit cycles, we haven’t labelled them. However, all limit cycles can be clearly seen in Fig 15. The simulated EEG for some stable limit cycles are plotted in Fig 15T_1_–15T_6_ (the initial conditions and the corresponding values of the input *u* for all times series are provided in Appendix B). We also observed that the simulated trajectory of the network for unstable limit cycles converges to either the branch of equilibria in Fig 14A and 14D or the limit cycle LC_14,2_. Furthermore, we found a limit cycle that appears from the saddle-node homoclinic bifurcation of equilibria at SN_14,3_. This orbit provokes spike-wave-like discharges in region *a* and periodic output that is alpha-like with some amplitude modulation, as plotted in Fig 15T_5_ and 15T_6_.

**Table 8:** The values of input *u* at bifurcation points in Figs 14 and 15.

Bifurcation	H_14,1_	H_14,2_	H_14,3_	SN_14,1_	SN_14,2_
Values of *u*	-12.15	89.83	315.7	113.6	136.4
Bifurcation	H_14,4_	H_14,5_	H_14,6_	SS_14,1_	
Values of *u*	-12.15	89.83	315.7	113.6	
Bifurcation	H_14,7_	H_14,8_	H_14,9_	SN_14,3_	
Values of *u*	-12.15	89.83	315.7	113.6	

**Fig 16:**
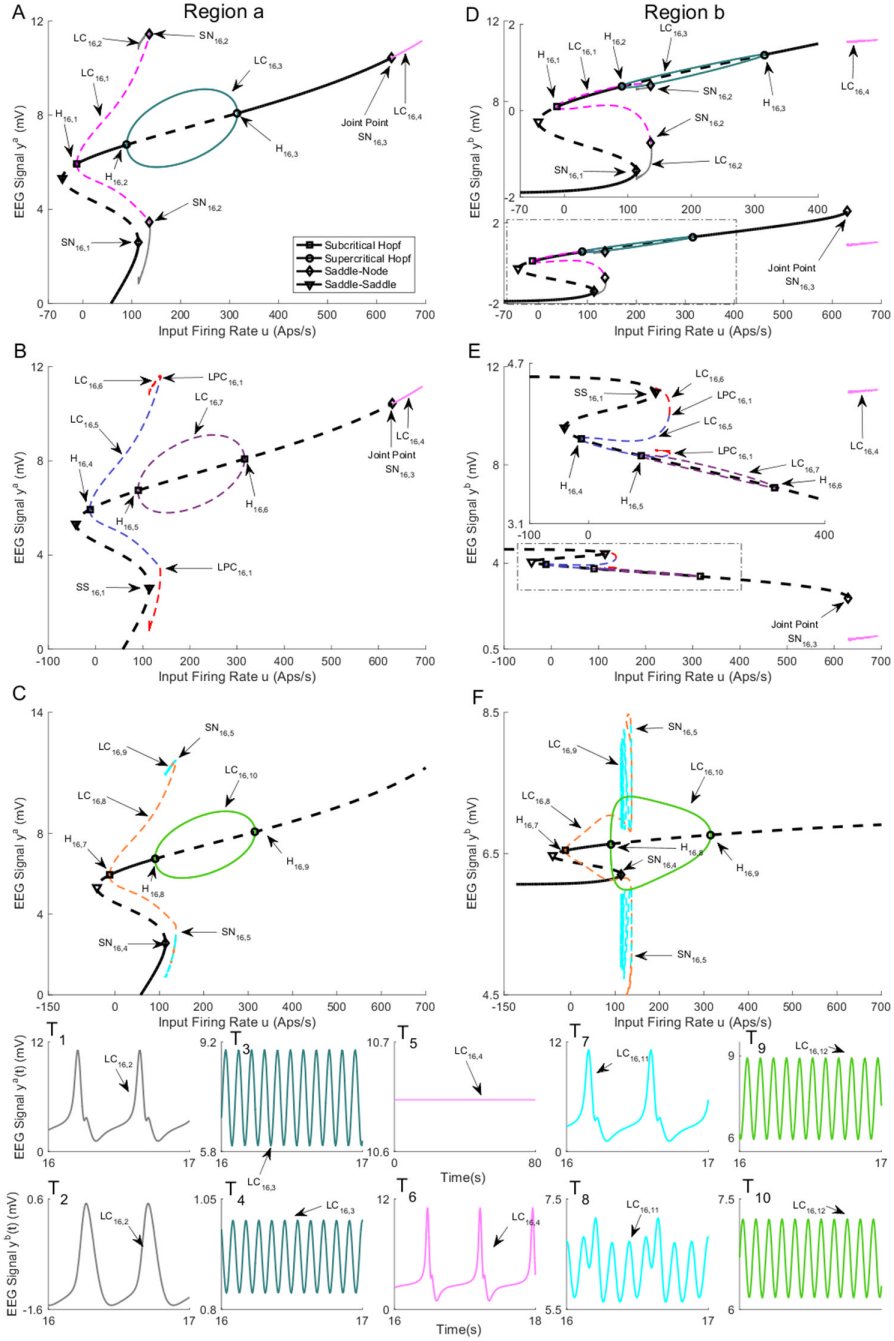
Bifurcation diagrams for case III with coupling gain *K* = 250. A), B), and C) are the first, second, and the third branches of equilibria for region *a*. D), E), and F) are the first, second, and the third branches of equilibria for region *b*. Panels T_1_-T_10_) show the EEG time series corresponding to the each part in the bifurcation diagrams. The solid black lines show stable fixed points, the solid colored lines show stable oscillatory behavior and the dashed lines show unstable fixed points and unstable oscillations. The initial conditions and the corresponding values of the input *u* for all times series are provided in Appendix B.

The bifurcation diagrams for coupling gain *K* = 250 are plotted in Fig 16. The values of *u* for all bifurcation points are presented in Table 9. Similar to case II with coupling gain *K* = 250, there is a limit cycle LC_16,4_ that starts from a saddle-node homoclinic bifurcation SN_16,3_ on the first branch of equilibria for *u* = 631. As shown in Fig 16T_5_ and 16T_6_, region *b* and region *a* show spike-wave-like discharges with the frequency of 1.25Hz and constant behavior in the time domain, respectively, when the whole network evolves on the cycle (the initial conditions and the corresponding values of the input *u* for all times series are provided in Appendix B). The bifurcation diagram of the second branch of equilibria in Fig 16C and 16F includes a stable limit cycle LC_16,10_, results from a supercritical Hopf bifurcation, and generates alpha-like activity in both regions (Fig 16T_9_ and 16T_10_). The unstable limit cycle LC_16,8_ collides with the limit cycle LC_16,9_, resulting from the saddle-node homoclinic bifurcation, for *u* = 135.4. According to the simulated EEG in Fig 16T_7_ and 16T_8_, the limit cycle LC_16,9_ results in the appearance of spikes with the frequency of 3Hz in the region *a* and delta-like output in the region *b*.

**Table 9:** The values of input *u* at bifurcation points in Fig 16.

Bifurcation	H_16,1_	H_16,2_	H_16,3_	SN_16,1_	SN_16,2_
Values of *u*	-12.15	89.83	315.7	113.6	136.4
Bifurcation	H_16,4_	H_16,5_	H_16,6_	SS_16,1_	SN_16,3_
Values of *u*	-12.15	89.83	315.7	113.6	629.5
Bifurcation	H_16,7_	H_16,8_	H_16,9_	SN_16,4_	SN_16,5_
Values of *u*	-12.15	89.83	315.7	113.6	135.4

### 2.4 Relationship to clinical data

We have presented a series of snapshot bifurcation diagrams to explore different behaviors that can be observed in interconnected neural mass models. In this section, we relate our analyses to clinical ECoG recorded from two electrode channels during seizures from a single patient with refractory temporal lobe epilepsy. Data was obtained from a previous clinical trial (see [32] for details, the current patient is subject 3. In the current estimation, two focal electrode channels were selected based on the signal energy at seizure onset. Electrodes were 5 mm in diameter, and the two channels were separated on the order of centimeters. The coupled Jansen and Rit model from this work has been theorized to describe EEG/MEG activity [11]; hence, is suitable for ECoG measured at this scale. State and parameter estimation were conducted on two 6 minute recordings (sampled at 400 Hz), each containing a different epileptic seizure. The estimation approach used a method of Gaussian belief propagation (see Appendix D for detail on the estimation method) to simultaneously track fast states (the membrane potentials of the population in the coupled neural mass model and their derivatives), the slowly varying bifurcation parameter *u* (representing the external input to each neural region), and a DC offset to compensate for drift introduced by changes in the input parameter (since the data had previously been amplified using a common average reference, removing most true DC content from the signal). We first estimated the parameter *u* from data using the assumed density filter. We then performed forward numerical integration of the model states using the estimated values for *u* and keeping all other values fixed. Simulation provides further insight into the predicted dynamics of the output ECoG based on alterations in input.

The estimation proceeded as follows, data were first pre-processed using a zero-phase bandpass (1-180 Hz) and notch filter (50Hz notch), and upsampled (lowpass interpolation) to 1200 Hz. Data were also scaled to reflect the dynamic range observed in the bifurcation analysis (approximately 0—12 mV). The estimation algorithm has three steps; initialization, prediction, and update. Initialization sets the estimation prior as a multivariate Gaussian probability density function (pdf) over the estimation states and parameters. The next step is to predict the posterior pdf by propagating the Gaussian prior through the non-linear, discretized neural mass equations. The update step then adjusts the predicted posterior based on the incoming measurement. Finally, the prior is reinitialized as a Gaussian distribution with the same mean and variance as the posterior, and the process is iterated for the next time step ($dt=\frac{1}{1200}$). In the case of a linear model, this estimation scheme is known as the Kalman filter [33]; however, here we are able to use a fast, semi-analytic solution to the belief propagation step to remove the linearity assumption. Unlike sampling based approximations, such as the unscented Kalman filter [34], our estimation method provides a precise solution for belief propagation. Nevertheless, several simplifying assumptions are used; model and measurement errors are described by additive, white Gaussian noise, and cortical dynamics are assumed to be Markovian, or memoryless. These assumptions are certainly not ideal for modeling epileptic dynamics; however, without this simplification there is no tractable solution for tracking parameters in real time. To our knowledge this algorithm reflects the current state-of-the-art for joint state and parameter estimation in the neural mass model [28], and the best available solution for relating measured ECoG to the hidden bifurcation parameter *u*.

The following sections relate the estimation results for the bifurcation parameter *u* in each of the three models (Case I, II, and III) for weak coupling (*K* = 50) to the dynamic snapshots that were presented in the preceding sections. The estimation is the statistically most likely evolution of the input parameter *u* given a distribution conditioned jointly on the model parameters and data (and subject to the assumptions outlined above). In addition to performing estimation, we also implemented a deterministic forward simulation of the coupled regions using the Runge-Kutte method on the discretized form of Eq 1. We first estimated the parameter *u* from data using the assumed density filter. We then performed forward numerical integration of the model states, using the estimated values for *u* and keeping all other parameter values fixed (according to Table 1). Simulation provides further insight into the predicted dynamics of the output ECoG based on alterations in input. All code used for estimation and simulation was implemented in MATLAB and Statistics Toolbox (release 2015a, The MathWorks Inc., MA, United States) and is available online from https://github.com/pkaroly/Bifurcation-Estimation.

#### 2.4.1 Seizure one

**Fig 17:**
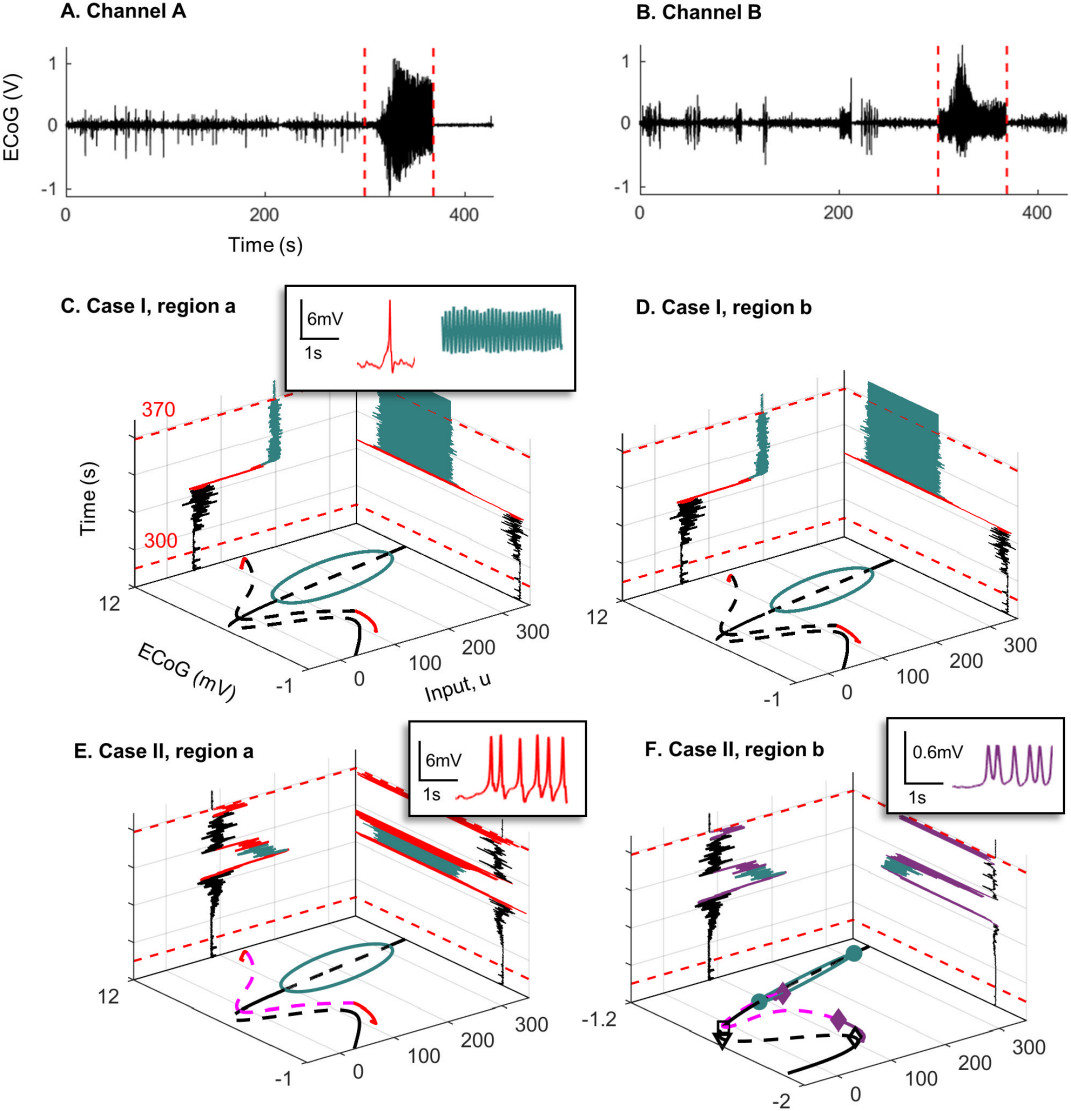
Recorded ECoG from two channels for seizure one and the bifurcation diagrams. Panels A) and B) are ECoG recordings of the same seizure (seizure one) on two different electrode channels. Recording was taken five minutes prior to seizure onset (red dashed line) and continued for 1 minute after offset (red dashed line). C) and D) show the bifurcation diagrams corresponding to case I, estimated input parameter *u* during the seizure (left wall panel) and the output ECoG after forward simulation based on the estimated input (right wall panel). Note that the plot only shows estimation from 10s before seizure onset to 10 s after seizure offset. E) and F) show the same plots as C) and D) but correspond to case II of the coupled neural mass model. Insets show the different waveforms (color-coded) that were found during forward simulation (right wall panel).

Fig 17A and 17B show recorded ECoG from two channels for seizure one (data were sampled at 400 Hz and bandpass filtered between 1—180 Hz). For interconnected model in the Case I, the estimation results are plotted on the left wall panel of Fig 17C and 17D. These figures indicate that, during the early stage of the seizure, the estimated input *u* varies between 80 and 100 followed by a sudden increase to approximately 200. By comparing the estimated parameter to the bifurcation plot in the floor panel of Fig 17C and 17D, we see that, for the first range of the input, the system has two orbits which are associated with spike generation. However, the bifurcation diagrams show that by increasing the input, the system transitions to the limit cycle. There is no clear transition at the end of the seizure (red dashed line) as the model does not transition back to a fixed point within a 10s period following seizure termination. Consequently, if the estimated input is applied to the model in forward simulation (with all other parameters fixed), it will show alpha activity at the end of seizure, as we see in the right wall panel of Fig 17C and 17D. The discrepancy between the predicted output and actual ECoG results in a large estimation error. The filter covariance is proportional to prediction error, so the estimated parameters will eventually adjust to better reflect the data; however, in this case, adjustment is not fast enough to capture the transition out of the seizure. Therefore, this model configuration may not be suitable to capture the observed behavior for seizure one, where there was a clear transition in the ECoG waveform at seizure termination (see Fig 17A and 17B)).

The estimation process yielded a similar range of inputs for cases II and III, suggesting that the transition between normal behavior and epileptic activity mainly results from the first branch of equilibria, since the bifurcation diagram associated to the first branch of equilibria for case II (Fig 9A and 9D) is the same as case III (Fig 14A and 14D). The estimation results in the left wall panel of Fig 17E and 17F show that, early in the seizure, the input varies between 80 and 90, then briefly reaches a peak around 200, approximately 40 s into the seizure. This input peak pushes the trajectory of the system into the limit cycle after some transient spiking possibly caused by the orbit originating from the saddle-node homoclinic bifurcation. The transition in region *a* also drives region *b* to transition into a limit cycle. This transition corresponds to the seizure reaching its peak amplitude on the ECoG in Channel A (Fig 17A). However, as the input drops to the range of 90 to 100, the system state is attracted once more to the cycle, and returns to epileptiform spiking activity in region *a* and amplitude modulated alpha activity in region *b*, before returning to a fixed point.

#### 2.4.2 Seizure two

**Fig 18:**
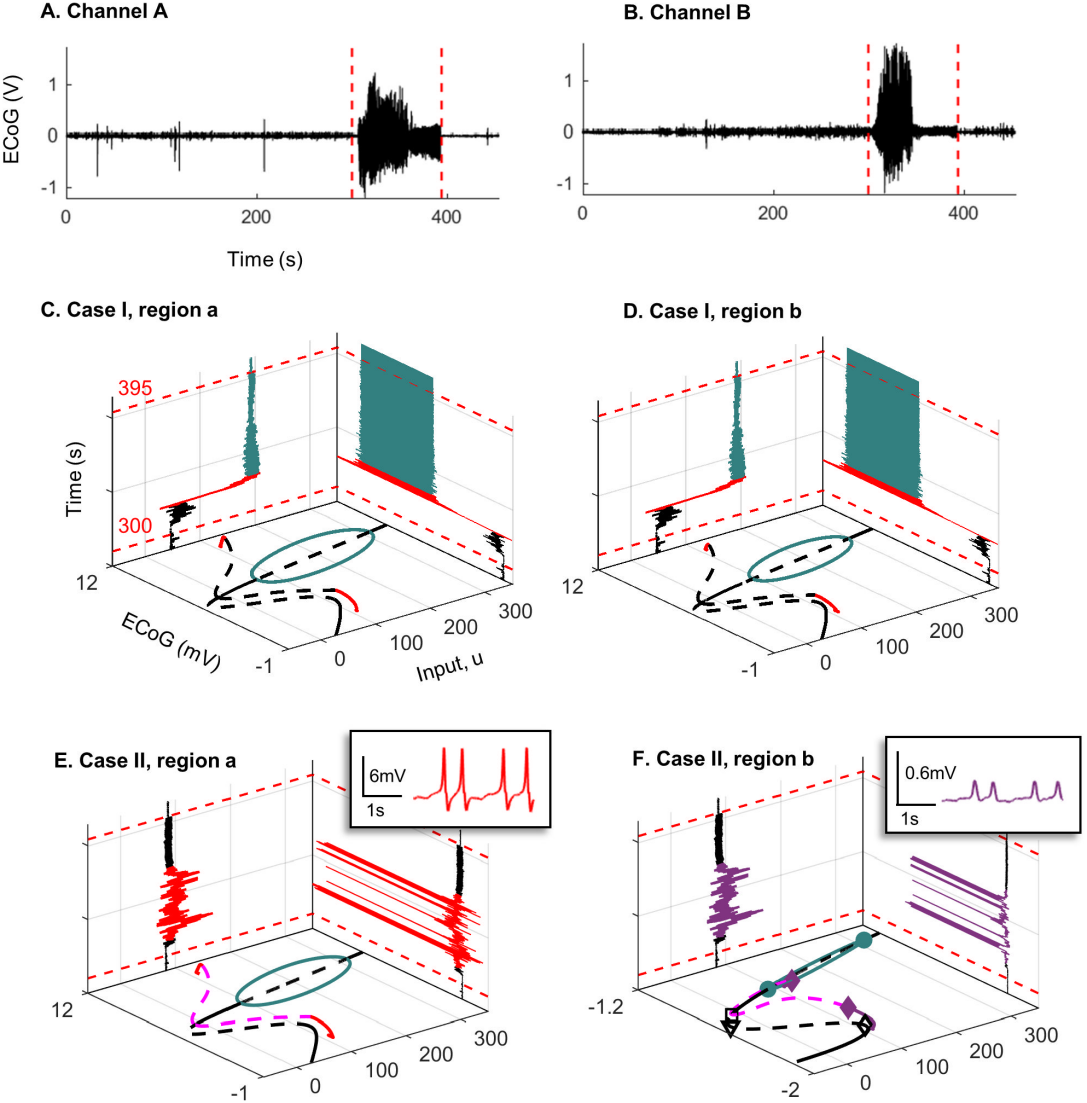
Recorded ECoG from two channels for seizure two and the bifurcation diagrams. Panels A) and B) are ECoG recordings of the same seizure (seizure two) on two different electrode channels. Recording was taken five minutes prior to seizure onset (red dashed line) and continued for 1 minute after offset (red dashed line). C) and D) show the bifurcation diagrams corresponding to case I, estimated input parameter *u* during the seizure (left wall panel), and the output ECoG after forward simulation based on the estimated input (right wall panel). Note that the plot only shows estimation from 10s before seizure onset to 10s after seizure offset. E) and F) show the same plots as C) and D) but correspond to case II of the coupled neural mass model. Insets show the different waveforms (color-coded) that were found during forward simulation (right wall panel).

Fig 18A and 18B show recorded ECoG for seizure two. The estimation results for Case I (Fig 18C and 18D) for seizure two are very similar to the previous seizure. However, Case II (Fig 18E and 18F) shows some differences to seizure one. The estimation for Case II was the same of that for Case III. Here, for Case II, the input is higher (*u* > 110) early in the seizure and continues to vary around this level. Conversely, during seizure one, the peak was higher (*u* > 200), and occurred later in the seizure (at approximately 40s). Following the peak in seizure one, the input dropped approximately monotonically. These lower yet sustained input peaks during seizure two indicate that the states of the system experienced more transients near the homoclinic orbit. Consequently, in the simulated ECoG obtained from the estimated input *u* (right wall panels of Fig 18E and 18F), we see epileptic spiking during the seizure only in region *a*. This is consistent with bifurcation analysis in Fig 9A and 9D in which only region *a* shows spikes. The difference between the results for the two data sets suggest that during some seizures region *b* is driven into a limit cycle, but during other seizures this state change does not occur. Interestingly, seizure two occurred in the middle of the day (around 1pm), whereas the first seizure occurred at night (approximately 10pm), so it is possible the different mechanisms were related to different states of arousal, although many more seizures would be required to investigate this hypothesis.

## 3 Discussion

This paper presented a bifurcation analysis of a neural mass model for two cortical regions. The results detail the rich repertoire of dynamics that the network can generate and how the range of possible activity varies with changes in the external inputs and interconnectivity gains. The bifurcation plots extend previous analyses of single region neural mass models and show that the dynamics of the interconnected neural masses can generate a far broader range of oscillatory dynamics, including multiple alpha-like rhythms, transient bursting, spikes, and delta wave activity.

Interestingly, for all cases and all interconnectivity gains, the models were able to produce alpha-like oscillation. Furthermore, in all of the scenarios that were explored, the alpha-like rhythms occurred concurrently in both regions or not at all. Similar to earlier studies considering a single region model [13, 20], the alpha rhythms were always generated by stable limit cycles originated from supercritical Hopf bifurcations. A key difference for the multiple region model is the existence of multiple types of alpha-like rhythms, representing different limit cycles with various amplitudes ranges. It is also interesting to note that network of identical regions with symmetric coupling and balanced inputs can generate oscillations with different amplitudes across the regions. The idea of the coexistence of multiple types of a discrete number of alpha rhythms builds on existing studies and should be investigated experimentally [35].

Our analysis revealed interesting insights into the possible mechanisms of the generation of spike-wave discharges. In case I of the symmetrical network with weak inter-region coupling, our results are naturally similar to existing results for a single neural mass model [20]. However, further important insights can be gained when studying two regions. As the coupling gain is increased, we see a merger of the outer limit cycle, which is responsible for the alpha-like rhythm, with the pathological orbit that is responsible for the generation of spike-wave like discharges. This merger of the respective limit cycles represents one candidate explanation for the process of epileptogenesis. Although, it should also be pointed that there are other candidate models for seizure transitions. In this work, for all values of connectivity gains, the model can transient from fixed points to orbit and vise versa. These transitions may also be the responsible model for seizure.

For Case II with high interconnection gains, the underlying network was able to generate spikes for values of input larger than a specific value as seen in Fig 12. This new result shows the networks with this structure can transition to an epileptic form pattern of activity given a sufficiently strong input, as the orbit, resulted from saddle-node homoclinic bifurcation, is the only stable pattern of activity. This finding contrasts other cases when a perturbation from other stable cycles may also be required. This network was able to generate alpha activity for values of input larger than a specific value as seen in Fig 12. This is also a new observation which shows that this structure can exhibit alpha activity for sufficiently strong input.

In Case III (Fig 16), we alarmingly see the occurrence of spike-wave discharges in region *b* (associated with the limit cycle LC_16,4_) due to increases in input to region *a*. The spikes do not occur in region *a*. The reason why this is alarming is that region *b* was set to represent background activity. Region *b* simply experienced a flow on effect from changes in the input to region *a* and is otherwise normal. This scenario poses a problem for planning epilepsy related surgery. The analysis shows that the presence of focal spike-wave discharges is not a sufficient condition to locate the pathology. The ideal treatment target in this scenario would be to limit the input in region *a*, as removal of region *b* would not treat the root cause.

The interconnected neural mass models are able to produce delta wave-like activity in Cases II and III. Interestingly, we observed stable delta wave-like activity in one region (Figs 9T_2_, 12T_2_, 14T_2_ and 16T_2_), and spike-wave-like activity in the other region (Figs 9T_1_, 12T_1_, 14T_1_ and 16T_1_). Delta activity was defined based on the frequency of oscillation (between 0.5-4Hz), and having a lower amplitude than epileptiform activity. The generation of delta-like activity may be linked to epileptiform spike generation in this model. Since the delta wave is observed during sleep, these networks can be potentially utilized to model and form a deeper understanding of nocturnal seizures in which a part of brain exhibits seizure activity while other parts do not. Our estimation results in Section 2.4 also suggested there are multiple mechanisms of seizures, which may correspond to different alertness levels. A more rigorous investigation estimating mechanisms of seizures at different times of day is the focus of ongoing work.

The estimation results showed that the model in Case I might not be representative of brain during and after the seizure. The estimated input could steer the model from spike-wave-like activity and a stable limit cycle; however, it was not able to transition back to a resting state. As a consequence, the estimated input did not drive the system to return to the pre-ictal state at the end of seizure. In contrast, the forward simulation using the estimated input showed that Case II and III could generate non-identical spikes in both regions, and also transition between spike-like activity to the pre-seizure behavior after the end of the seizures. Note that although the transition from a fixed point to a limit cycle arising from a Hopf bifurcation is referred to as ‘alpha’ activity, this class of transition is also used to describe seizure onset [36, 37]. The estimation results showed that the transition from a fixed point to a limit cycle occurred during seizures. For Case I, the failure to transition out of the limit cycle suggests that the models in Case II and III more closely capture seizure dynamics than Case I. We can speculate from this that once a seizure has spread, either an asymmetric, or possible alteration of the existing connectivity pattern is required for its termination. This is consistent with the analysis of [38], who suggest that a distinct bifurcation is required for seizure termination, compared to seizure onset.

Our estimation approach was conservative, as we estimated the input with other parameters fixed. By estimating more parameters, it may be possible to obtain a more realistic approximation of the true behavior. However, with more free parameters, it becomes difficult or impossible to relate the estimated parameter trajectories to a bifurcation analysis. Therefore, such an extension is beyond the scope of the current work. Nevertheless, our estimation is a qualitative picture of dynamical state changes from recorded ECoG, which may provide insight into mechanisms of seizures. For instance, we found that during seizure one, both regions were driven into the limit cycle, whereas in the second seizure this was not the case. Once the system enters a limit cycle, the pathologic state may be harder to terminate, due to a hysteresis effect whereby lowering *u* does not immediately reverse the effects of the transient increase (as shown in Fig 17E and 17F). Identifying such differences in seizure mechanisms is important for targeting treatment.

Before closing our discussion, it should be mentioned that the computational model is a crude approximation of a real brain. Nevertheless, it is challenging to present a more comprehensive model that describes a wide range of brain activities. The authors caution the reader to interpret the results as possible behaviors that can be generated from two interconnected cortical regions, rather than behaviors that will occur. Also, we stress that the range of possible dynamics holds for the two region model. Further increasing the complexity of the model by adding neural populations or cortical regions will undoubtedly yield a more complicated bifurcation structure. Nevertheless, the work can be regarded as a contribution, demonstrating the flexibility of this neural mass modeling framework.

As future work, this analysis can be extended by using co-dimension 2 bifurcation analysis with respect to both the interconnection gain and another network parameter. From a technical perspective, it is also valuable to analyze the geometric property of the limit cycles LC_12,4_ and LC_16,4_ that are born from the saddle-node homoclinic bifurcation in the first branch of equilibria in Cases II and III as the limit cycles LC_12,4_ generates spikes in both regions while the limit cycles LC_16,4_ generates spikes in only region *b*.

## A Detection of a saddle-node homoclinic bifurcation

**Fig 19:**
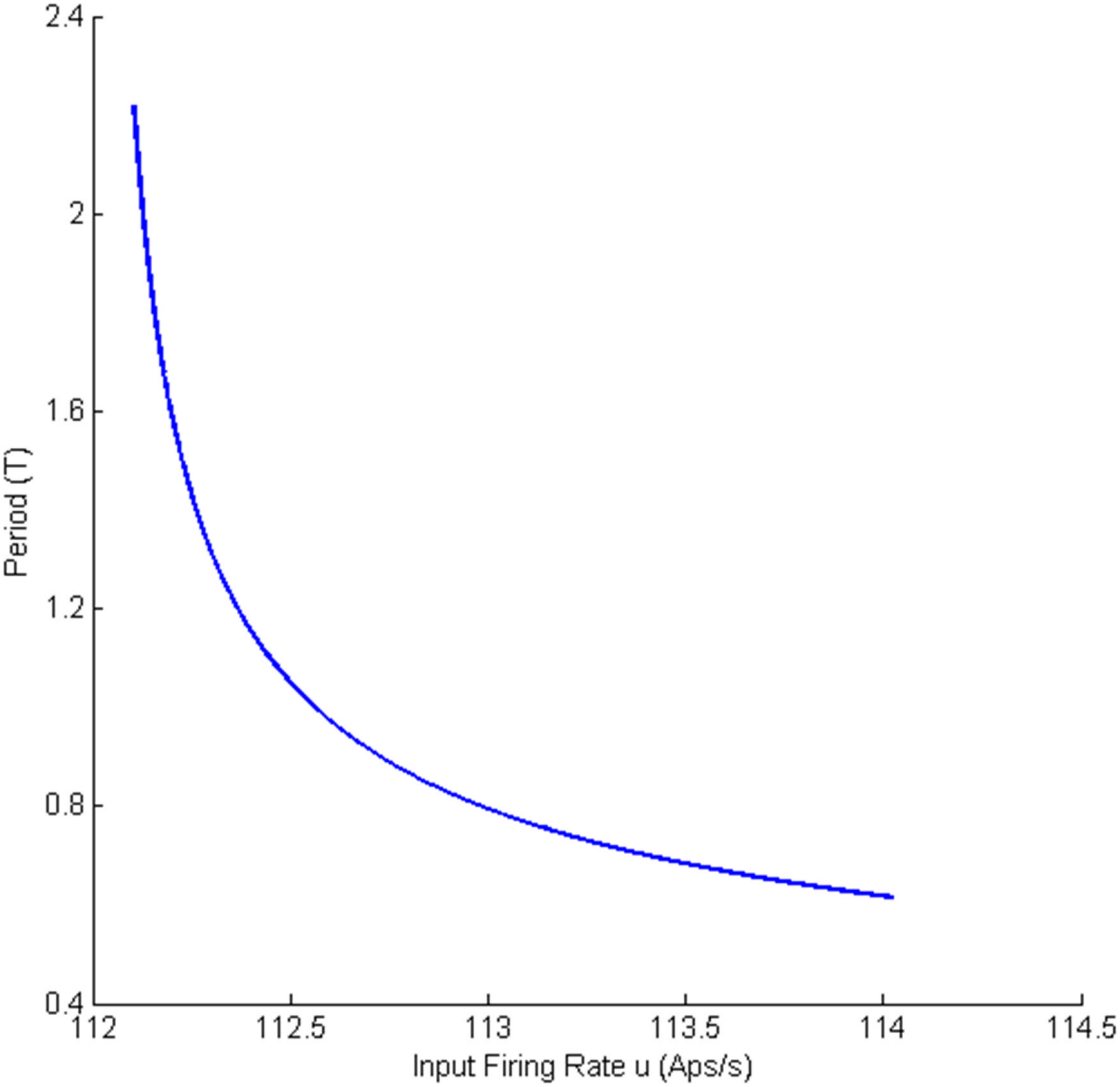
Detecting a saddle-node homoclinic bifurcation. The period of oscillation versus the bifurcation parameter p. The period eventually increases when the value of p approaches to the point at which saddle-node bifurcation occurs.

A homoclinic orbit is a trajectory connecting a hyperbolic equilibrium (saddle node) to itself. There is no general method to find and identify a limit cycle; however, it is possible to compute it using the continuation procedure provided in the MATCONT package [29]. In order to check if the bifurcation is saddle-node homoclinic, the period of oscillation versus the bifurcation parameters is usually plotted (it is depicted for case I with connection gain 25 in Fig 19). As the bifurcation parameter *u* approaches the bifurcation point, the period of oscillation is eventually increased (diverges to infinity) which means that the cycle is born from this point.

## B Initial conditions for time series depicted in bifurcation diagrams

The following tables provide initial conditions and the values of inputs that have been used to generate time series in all bifurcation diagrams.

Table 10 shows the initial conditions and the values of input for time series depicted in Fig 2T_1_–2T_3_.

**Table 10:** The initial conditions and the values of input for time series depicted in Fig 2T_1_–2T_3_.

Limit Cycle	$\left[v_{0}^{a},z_{0}^{a},v_{1}^{a},z_{1}^{a},v_{2}^{a},z_{2}^{a},v_{3}^{a},z_{3}^{a},v_{0}^{b},z_{0}^{b},v_{1}^{b},z_{1}^{b},v_{2}^{b},z_{2}^{b},v_{3}^{b},z_{3}^{b}\right]$	Input *u*
*LC*_2,1_	[0.08 -1.33 18.44 -10.83 13.35 10.36 0.28 -0.65 0.08 -1.33 18.44 -10.83 13.35 10.36 0.28 -0.65]	29.25
*LC*_2,2_	[0.08 0.93 16.95 36.44 9.89 -15.56 0.24 0.39 0.09 -1.13 18.39 -11.58 -13.12 6.40 0.28 -0.59]	29.17
*LC*_2,3_	[0.07 1.87 16.14 131.21 8.40 -48.23 0.21 0.51 0.09 -2.76 21.36 -10.78 17.48 -2.11 0.31 -1.51]	116.15
*LC*_2,4_	[0.08 -2.82 21.41 -11.24 17.68 -10.12 0.31 -1.55 0.08 -2.82 21.41 -11.24 17.68 -10.12 0.31 -1.55]	116.06
*LC*_2,5_	[0.10, -1.41, 24.21, -3.82, 18.32, -10.71, 0.35, -0.97, 0.12, 1.16, 24, 10.87, 14.91, 7.81, 0.32, 0.88]	198.7
*LC*_2,6_	[0.12 1.01 24.14 7.56 15.28 6.42 0.33 0.76 0.12 1.01 24.14 7.56 15.28 6.42 0.33 0.76]	201.65

Table 11 shows the initial conditions and the values of input for time series depicted in Fig 4T_1_ and 4T_2_

**Table 11:** The initial conditions and the values of input for time series depicted in Fig 4T_1_ and 4T_2_.

Limit Cycle	$\left[v_{0}^{a},z_{0}^{a},v_{1}^{a},z_{1}^{a},v_{2}^{a},z_{2}^{a},v_{3}^{a},z_{3}^{a},v_{0}^{b},z_{0}^{b},v_{1}^{b},z_{1}^{b},v_{2}^{b},z_{2}^{b},v_{3}^{b},z_{3}^{b}\right]$	Input *u*
*LC*_4,7_	[0.09 -0.25 20.26 -2.47 13.77 -1.23 0.30 -0.17 0.01 0.0005 4.56 0.05 3.09 -0.0035 0.03 0.0002]	90.50

Table 12 shows the initial conditions and the values of input for time series depicted in Fig 7T_1_–7T_5_.

**Table 12:** The initial conditions and the values of input for time series depicted in Fig 7T_1_–7T_5_.

Limit Cycle	$\left[v_{0}^{a},z_{0}^{a},v_{1}^{a},z_{1}^{a},v_{2}^{a},z_{2}^{a},v_{3}^{a},z_{3}^{a},v_{0}^{b},z_{0}^{b},v_{1}^{b},z_{1}^{b},v_{2}^{b},z_{2}^{b},v_{3}^{b},z_{3}^{b}\right]$	Input *u*
*LC*_7,1_	[0.07 1.10 15.37 61.67 8.60 -35.33 0.21 0.18 0.07 1.10 15.37 61.67 8.60 -35.33 0.21 0.18]	29.14
*LC*_7,2_	[0.04 -0.73 17.34 -294.26 14.21 -225.55 0.24 -2.56 0.02 0.02 0.38 7.09 44.32 3.43 9.27 0.062 0.41]	116.08
*LC*_7,3_	[0.09 0.37 17.83 9.76 11.13 -0.32 0.26 0.23 0.08 -0.38 18.14 -7.04 12.18 -1.03 0.27 -0.24]	5.86
*LC*_7,4_	[0.12 0.64 24.99 2.63 16.52 5.01 0.34 0.48 0.12 0.64 24.99 2.63 16.52 5.01 0.34 0.48]	198.90
*LC*_7,5_	[0.10 -1.58 25.14 -3.78 19.3 -11.45 0.35 -1.073 0.12 1.24 25.01 11.18 15.46 9.73 0.33 0.97]	201.94

Table 13 shows the initial conditions and the values of input for time series depicted in Fig 8T_1_–8T_4_.

**Table 13:** The initial conditions and the values of input for time series depicted in Fig 8T_1_–8T_4_.

Limit Cycle	$\left[v_{0}^{a},z_{0}^{a},v_{1}^{a},z_{1}^{a},v_{2}^{a},z_{2}^{a},v_{3}^{a},z_{3}^{a},v_{0}^{b},z_{0}^{b},v_{1}^{b},z_{1}^{b},v_{2}^{b},z_{2}^{b},v_{3}^{b},z_{3}^{b}\right]$	Input *u*
*LC*_8,1_	[0.06 0.87 13.79 55.37 7.67 -38.17 0.19 -0.07 0.06 0.87 13.79 55.37 7.67 -38.17 0.19 -0.07]	29.01
*LC*_8,2_	[0.03 -0.81 16.47 -308.04 13.72 -220.95 0.23 -2.61 0.02 0.3 6.98 44.38 3.35 11.62 0.05 0.50]	102.07
*LC*_8,3_	[0.11 -0.37 24.28 0.87 17.25 -2.32 0.34 -0.26 0.11 -0.37 24.28 -0.87 17.25 -2.32 0.34 -0.26]	158.18
*LC*_8,4_	[0.11 -1.64 25.67 -3.79 19.82 -11.58 0.36 -1.11 0.12 1.26 25.61 11.01 15.83 10.92 0.33 0.99]	200.94

Table 14 shows the initial conditions and the values of input for time series depicted in Fig 9T_1_–9T_10_.

**Table 14:** The initial conditions and the values of input for time series depicted in Fig 9T_1_–9T_10_.

Limit Cycle	$\left[v_{0}^{a},z_{0}^{a},v_{1}^{a},z_{1}^{a},v_{2}^{a},z_{2}^{a},v_{3}^{a},z_{3}^{a},v_{0}^{b},z_{0}^{b},v_{1}^{b},z_{1}^{b},v_{2}^{b},z_{2}^{b},v_{3}^{b},z_{3}^{b}\right]$	Input *u*
*LC*_9,2_	[0.04 -0.62 16.83 -277.72 13.59 -217.71 0.23 -2.47 0.002 0.001 1.15 -2.26 2.59 0.23 0.007 0.01]	119.02
*LC*_9,3_	[0.10 -1.31 23.99 -3.62 18.03 -9.30 0.34 -0.90 0.002 0.004 1.27 0.39 2.61 -0.004 0.007 0.0005]	200
*LC*_9,5_	[0.02 0.15 6.49 20.80 3.47 5.64 0.05 0.20 0.05 -0.002 10.58 0.07 6.05 0.28 0.15 0.008]	119.02
*LC*_9,6_	[0.10 -1.33 23.68 -3.74 17.78 -7.067 0.34 -0.89 0.05 0.004 10.19 0.18 5.79 -0.13 0.14 -0.0002]	183.92
*LC*_9,18_	[0.10 -1.33 23.79 -3.67 17.88 -7.13 0.34 -0.89 0.08 0.03 17.36 0.17 11.13 -1.69 0.26 -0.0008]	181.45

Table 15 shows the initial conditions and the values of input for time series depicted in Fig 12T_1_–12T_10_.

**Table 15:** The initial conditions and the values of input for time series depicted in Fig 12T_1_–12T_10_.

Limit Cycle	$\left[v_{0}^{a},z_{0}^{a},v_{1}^{a},z_{1}^{a},v_{2}^{a},z_{2}^{a},v_{3}^{a},z_{3}^{a},v_{0}^{b},z_{0}^{b},v_{1}^{b},z_{1}^{b},v_{2}^{b},z_{2}^{b},v_{3}^{b},z_{3}^{b}\right]$	Input *u*
*LC*_12,2_	[0.04 -0.64 17.71 -270.42 14.23 -218.83 0.24 -2.44 0.006 0.03 3.17 -7.06 2.71 2.59 0.01 0.13]	119.04
*LC*_12,3_	[0.10 -1.32 24.26 -3.41 18.27 -9.15 0.34 -0.90 0.009 0.02 3.93 2.19 2.93 -0.11 0.02 0.007]	201.93
*LC*_12,4_	[0.15 -0.0001 38.26 0.12 27.75 0.06 0.45 0.0004 0.02 0.0061 6.34 0.97 3.67 0.36 0.06 0.01]	621.40
*LC*_12,16_	[0.11 -1.43 26.13 -4.71 19.99 -7.09 0.36 -0.94 0.09 0.47 20.02 -0.21 12.99 -33.89 0.28 -0.05]	192.06
*LC*_12,17_	[0.13 -0.14 32.56 -2.21 23.89 -17.65 0.41 -0.21 0.10 0.85 20.40 11.63 12.69 -8.20 0.29 0.49]	389.46

Table 16 shows the initial conditions and the values of input for time series depicted in Fig 13T_1_–13T_18_.

**Table 16:** The initial conditions and the values of input for time series depicted in Fig 13T_1_–13T_18_.

Limit Cycle	$\left[v_{0}^{a},z_{0}^{a},v_{1}^{a},z_{1}^{a},v_{2}^{a},z_{2}^{a},v_{3}^{a},z_{3}^{a},v_{0}^{b},z_{0}^{b},v_{1}^{b},z_{1}^{b},v_{2}^{b},z_{2}^{b},v_{3}^{b},z_{3}^{b}\right]$	Input *u*
*LC*_12,8_	[0.07 -2.70 21.57 -27.52 18.01 -57.76 0.30 -1.84 0.09 0.19 19.57 11.39 12.67 13.02 0.28 0.28]	59.98
*LC*_12,9_	[0.08 0.08 16.99 0.28 10.80 -5.53 0.25 -0.01 0.09 -0.49 19.32 -6.04 13.26 -0.50 0.28 -0.31]	−62.16
*LC*_12,10_	[0.07 0.13 15.63 0.45 9.70 -10.09 0.23 -0.04 0.08 -1.28 19.22 -14.52 14.03 -6.56 0.29 -0.83]	−85.21
*LC*_12,11_	[0.07 0.09 14.70 0.44 9.006 -6.67 0.22 -0.03 0.08 -1.34 19.10 -15.88 14.002 -8.49 0.28 -0.88]	−99.15
*LC*_12,12_	[0.04 0.02 10.12 0.83 5.74 -14.13 0.14 -0.01 0.08 -1.13 18.36 -17.69 13.19 -8.70 0.27 -0.75]	−46.69
*LC*_12,13_	[0.01 0.02 4.73 2.97 3.11 -0.02 0.03 0.01 0.08 -0.70 17.33 -16.38 11.89 -5.06 0.26 -0.45]	37.04
*LC*_12,14_	[0.05 -1.68 22.09 -145.71 18.27 -191.73 0.259 -2.65 0.04 0.08 12.47 -63.82 7.99 -83.25 0.18 -1.10]	111
*LC*_12,15_	[0.07 -2.58 22.48 -39.38 18.67 -77.60 0.32 -2.15 0.05 0.57 15.51 -24.87 9.78 -101.38 0.22 -0.82]	95.75
Remark 1	[0.02 0.03 6.57 2.72 3.76 -0.54 0.06 0.001 0.08 -0.83 17.66 -17.09 12.30 -6.17 0.26 -0.54]	49.23

Table 17 shows the initial conditions and the values of input for time series depicted in Fig 14T_1_–14T_6_.

**Table 17:** The initial conditions and the values of input for time series depicted in Fig 14T_1_–14T_6_.

Limit Cycle	$\left[v_{0}^{a},z_{0}^{a},v_{1}^{a},z_{1}^{a},v_{2}^{a},z_{2}^{a},v_{3}^{a},z_{3}^{a},v_{0}^{b},z_{0}^{b},v_{1}^{b},z_{1}^{b},v_{2}^{b},z_{2}^{b},v_{3}^{b},z_{3}^{b}\right]$	Input *u*
*LC*_14,2_	[0.04 -1.02 19.79 -220.68 16.01 -2.06.03 0.26 -2.43 0.002 0.004 1.16 -0.73 2.59 0.24 0.006 0.01]	128.08
*LC*_14,3_	[0.10 -1.31 24.09 -3.51 18.11 -9.20 0.34 -0.90 0.002 0.001 1.27 0.39 2.61 -0.004 0.007 0.0005]	203.29
*LC*_14,7_	[0.11 1.11 23.73 10.07 14.82 5.76 0.32 0.82 0.08 -0.03 17.37 -0.12 11.17 1.64 0.26 0.0002]	199.30

Table 18 shows the initial conditions and the values of input for time series depicted in Fig 15T_1_–15T_6_.

**Table 18:** The initial conditions and the values of input for time series depicted in Fig 15T_1_–15T_6_.

Limit Cycle	$\left[v_{0}^{a},z_{0}^{a},v_{1}^{a},z_{1}^{a},v_{2}^{a},z_{2}^{a},v_{3}^{a},z_{3}^{a},v_{0}^{b},z_{0}^{b},v_{1}^{b},z_{1}^{b},v_{2}^{b},z_{2}^{b},v_{3}^{b},z_{3}^{b}\right]$	Input *u*
*T*_1_	[0.08 2.73 15.10 302.19 5.52 71.07 0.15 2.71 0.08 0.04 16.93 4.84 10.72 5.49 0.25 0.08]	137.23
*T*_3_	[0.03 0.29 8.21 43.51 4.14 8.95 0.08 0.42 0.08 0.21 16.67 11.40 10.32 8.27 0.24 0.22]	115.45
*T*_5_	[0.03 0.29 8.26 43.30 4.17 8.72 0.08 0.41 0.083 -0.04 16.83 -2.09 10.79 -2.26 0.25 -0.05]	114.22

Table 19 shows the initial conditions and the values of input for time series depicted in Fig 16T_1_–16T_10_.

**Table 19:** The initial conditions and the values of input for time series depicted in Fig 16T_1_ and 16T_10_.

Limit Cycle	$\left[v_{0}^{a},z_{0}^{a},v_{1}^{a},z_{1}^{a},v_{2}^{a},z_{2}^{a},v_{3}^{a},z_{3}^{a},v_{0}^{b},z_{0}^{b},v_{1}^{b},z_{1}^{b},v_{2}^{b},z_{2}^{b},v_{3}^{b},z_{3}^{b}\right]$	Input *u*
*LC*_16,2_	[0.03 -0.62 16.61 -277.80 13.42 -216.96 0.23 -2.47 0.006 0.01 3.13 -9.38 2.72 2.56 0.01 0.12]	119
*LC*_16,3_	[0.10 -1.31 24.17 -3.42 18.18 -9.12 0.34 -0.89 0.008 0.01 3.92 2.17 2.92 -0.11 0.02 0.007]	205.87
*LC*_16,4_	[0.15 0 38.67 0 28.02 0 0.45 0 0.03 0.24 8.03 35.61 4.11 7.52 0.08 0.35]	650
*LC*_16,11_	[0.01 -0.25 6.95 -91.17 5.77 -84.90 0.12 -1.86 0.07 -0.02 17.91 -45.75 12.03 -65.50 0.26 -0.73]	119
*LC*_16,12_	[0.09 -0.82 23.93 -9.67 17.72 -65.88 0.33 -1.13 0.09 0.52 19.85 2.71 12.68 -14.09 0.28 0.19]	200.35

## C Notes on equilibria for case II

As pointed out in Sections 1.3 and 2.2, the equilibria for the second case are obtained from (5), (6) and (7) with *u*^*b*^ = 0. In this case, (7) can be written as follows (*K*^*ab*^ = *K*^*ba*^ = *K*):

$$\begin{array}{c} \begin{array}{cc} y^{a}= & \frac{\alpha_{e}}{\zeta_{e}}c_{ep}g(\frac{\alpha_{e}}{\zeta_{e}}c_{pe}g\left(y^{a}\right))-\frac{\alpha_{i}}{\zeta_{i}}c_{ip}g(\frac{\alpha_{e}}{\zeta_{e}}c_{pi}g\left(y^{a}\right))+{\tilde{u}}^{a}\\ y^{b}= & \frac{\alpha_{e}}{\zeta_{e}}c_{ep}g(\frac{\alpha_{e}}{\zeta_{e}}c_{pe}g\left(y^{b}\right))-\frac{\alpha_{i}}{\zeta_{i}}c_{ip}g(\frac{\alpha_{e}}{\zeta_{e}}c_{pi}g\left(y^{b}\right))+{\tilde{u}}^{b}, \end{array} \end{array}$$ (9)

where

$$\begin{array}{c} \begin{array}{cc} {\tilde{u}}^{a} & =\frac{\alpha_{e}}{\zeta_{e}}u^{a}+\frac{\alpha_{e}^{2}}{\zeta_{e}\zeta_{d}}Kg\left(y^{b}\right)\\ {\tilde{u}}^{b} & =\frac{\alpha_{e}^{2}}{\zeta_{e}\zeta_{d}}Kg\left(y^{a}\right). \end{array} \end{array}$$ (10)

This implies that finding the equilibria of the whole network is equivalent to finding the equilibria of each single region when the input of each region are defined by (10).

We first claim that, the equilibria of region *a* are affected significantly by changing *u*^*a*^ rather than changing the values of *K* while the equilibria of region *b* are affected significantly by variations of *K*. Since the sigmoid function satisfies *g*(⋅) ≤ 2*e*_0_, the following inequalities are obtained from (10):

$$\begin{array}{c} \begin{array}{cc} {\tilde{u}}^{a} & \leq \frac{\alpha_{e}}{\zeta_{e}}u^{a}+\frac{2e_{0}\alpha_{e}^{2}}{\zeta_{a}\zeta_{d}}K\\ {\tilde{u}}^{b} & \leq \frac{2e_{0}\alpha_{e}^{2}}{\zeta_{a}\zeta_{d}}K. \end{array} \end{array}$$ (11)

For typical values of *e*_0_, *α*_*e*_, *ζ*_*a*_, and *ζ*_*d*_ (see Table 1), the value of $\frac{2e_{0}\alpha_{e}^{2}}{\zeta_{a}\zeta_{d}}$ is on the order of 10^−2^ and, consequently, the variation of $\frac{2e_{0}\alpha_{e}^{2}}{\zeta_{a}\zeta_{d}}K$ is much smaller than the variation of *u* even for large values of *K*. Hence, *y*^*a*^ and, consequently, the equilibria of region *a* are not affected significantly by feedback from region *b* due to the small interaction term $\frac{\alpha_{e}^{2}}{\zeta_{e}\zeta_{d}}Kg\left(y^{b}\right)$; however, the equilibria of region *b* are significantly affected by the output of region *a*.

In order to find the equilibria, we used a numerical approach to find all values for *y*^*a*^ and *y*^*b*^ which satisfy (9) and (10) for different values of *u*^*a*^ and *K*. To do so, we varied the value of *y*^*b*^ ∈ (−20, 20) and calculated the value of *y*^*a*^ from the second equation in (9), i.e. from solving the equation,

$$g\left(y^{a}\right)=\underset{ϒ\left(y^{b},K\right)}{\underset{︸}{\frac{\zeta_{e}\zeta_{d}}{K\alpha_{e}^{2}}\left(y^{b}-\frac{\alpha_{e}}{\zeta_{e}}c_{ep}g\left(\frac{\alpha_{e}}{\zeta_{e}}c_{pe}g\left(y^{b}\right)\right)+\frac{\alpha_{i}}{\zeta_{i}}c_{ip}g\left(\frac{\alpha_{e}}{\zeta_{e}}c_{pi}g\left(y^{b}\right)\right)\right)}}.$$ (12)

By knowing the value of *y*^*a*^ and *y*^*b*^, the associated value *u*^*a*^ was obtained from the first equation in (9), which can be rewritten as

$$\begin{array}{c} \begin{array}{c} u^{a}=\frac{\zeta_{e}}{\alpha_{e}}(\frac{\alpha_{e}}{\zeta_{e}}c_{ep}g(\frac{\alpha_{e}}{\zeta_{e}}c_{pe}g\left(y^{a}\right))-\frac{\alpha_{i}}{\zeta_{i}}c_{ip}g(\frac{\alpha_{e}}{\zeta_{e}}c_{pi}g\left(y^{a}\right))+\frac{\alpha_{e}^{2}}{\zeta_{e}\zeta_{d}}Kg\left(y^{b}\right)-y^{a}). \end{array} \end{array}$$ (13)

Since *g*(*v*) is a strictly increasing function and 0 ≤ *g*(*v*) ≤ 2*e*_0_, Eq (12) has a solution if and only if

$$\begin{array}{c} 0\leq ϒ\left(y^{b},K\right)\leq 2e_{0}. \end{array}$$ (14)

**Fig 20:**
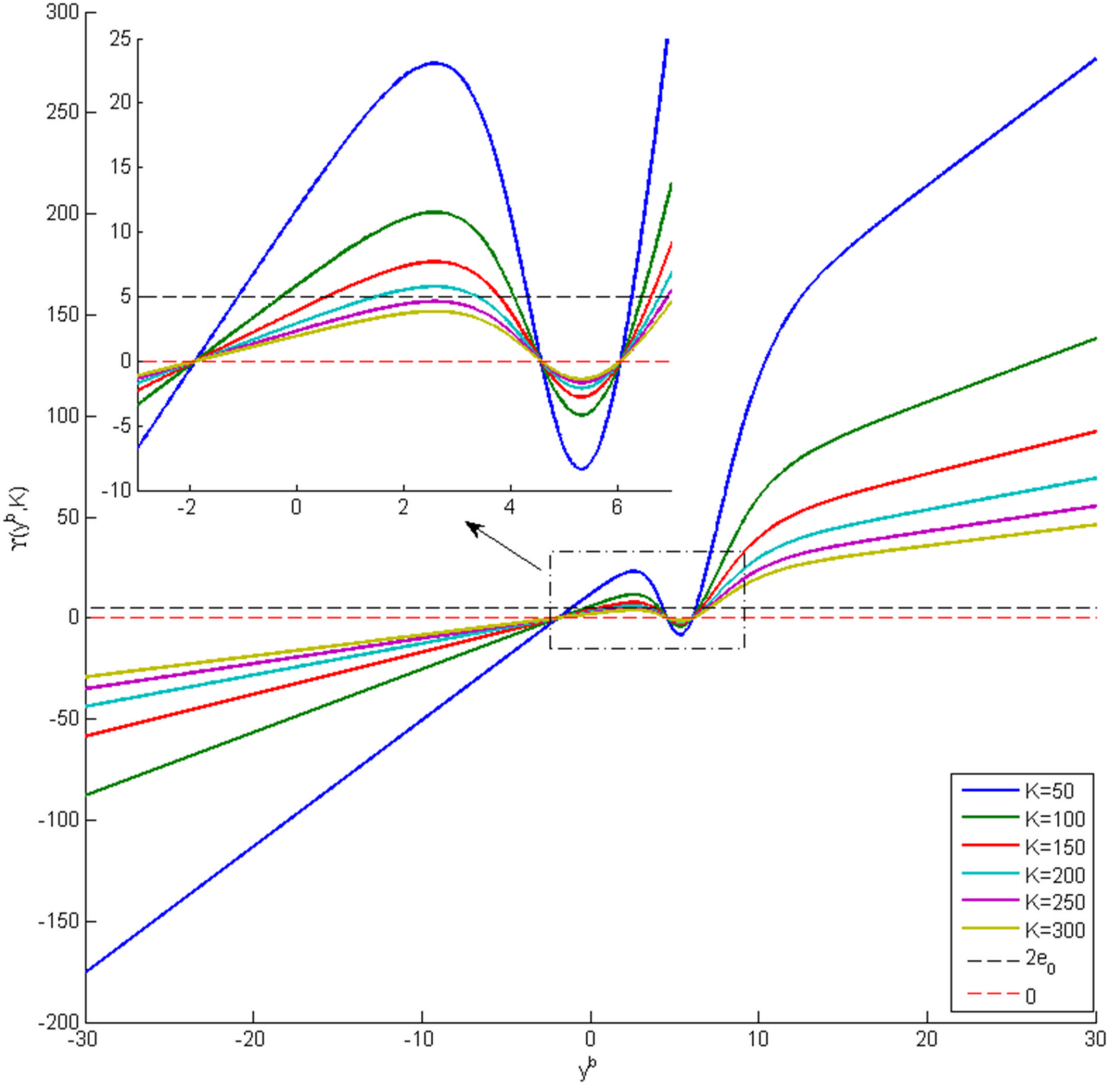
The values of ϒ(*y*^*b*^, *K*) for different values of coupling gain *K*.

**Fig 21:**
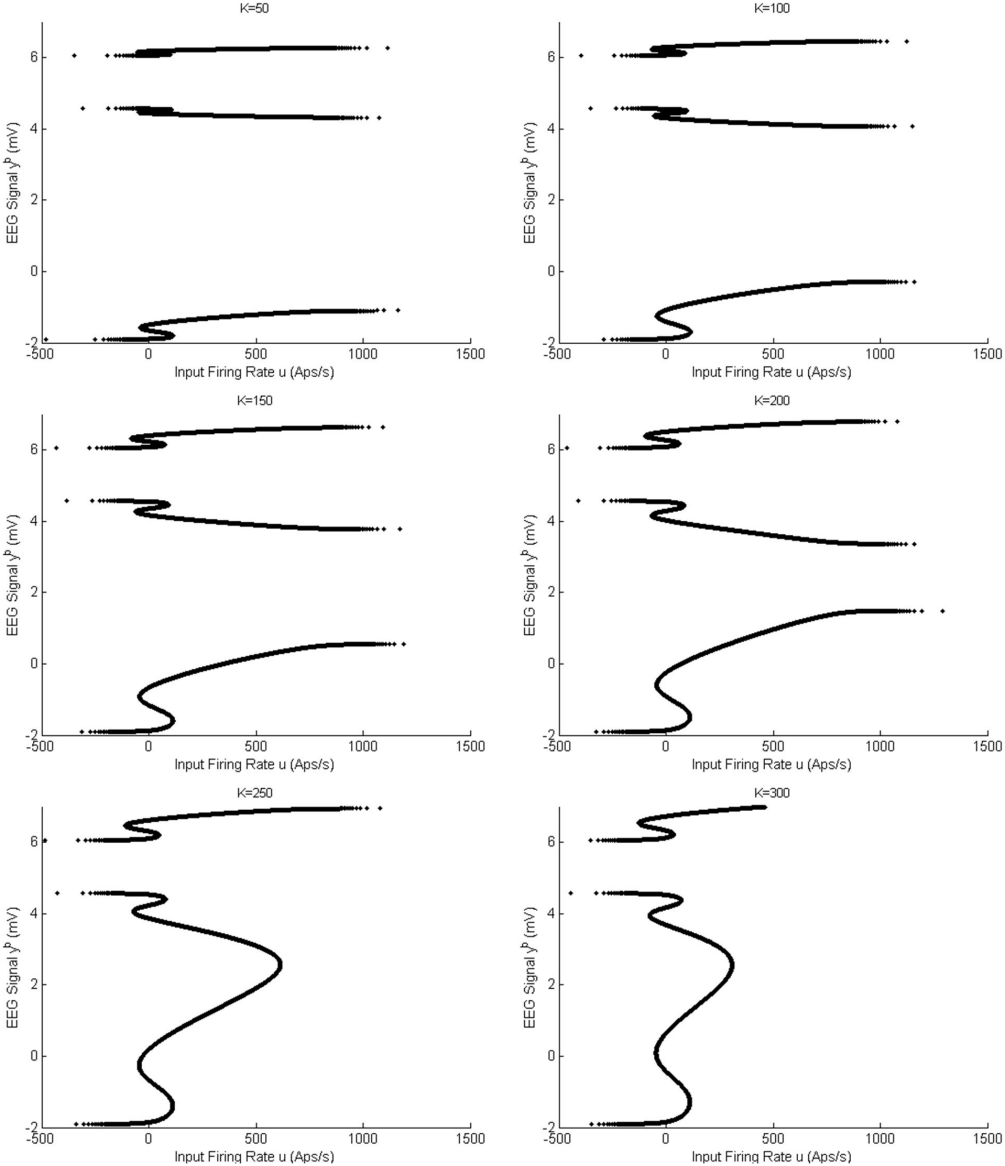
Equilibria of second region for case II and for different values of coupling gain *K*. In this figure, solid black lines show the equilibria regardless of their stability or instability. The three branches of equilibria for *K* = 50 are labeled according to Fig 9. The saddle-node for *K* = 250 is labeled according to Fig 12.

Among all values of *y*^*b*^ ∈ (−20, 20), the acceptable ones are those that satisfy (14). As a consequence, some values in the interval *y*_*b*_ ∈ (−20, 20) may not be equilibria. For typical values of *e*_0_, *α*_*e*_, *ζ*_*a*_, and *ζ*_*d*_ (see Table 1), we plotted ϒ(*y*^*b*^, *K*) for *y*^*b*^ ∈ (−30, 30) and different values of *K* in Fig 20. This figure indicate that the inequality (14) cannot be satisfied fur sufficiently large and small values of *y*^*b*^ that means that there exist no equilibria for those values of *y*^*b*^. From the magnified part of the figure, it is observed that, for all values of *K*, there is no equilibrium point for *y*^*b*^ ∈ (4.57, 6.07). Furthermore, the underlying network has equilibria for all values for *y*^*b*^ ∈ (−1.9, 4.57) if *K* = 250, 300. However, this is not the case for *K* = 50, 100, 150, 200. This indicates that the second region has three branches of equilibria that do not intersect for *K* = 50, 100, 150, 200. This point can be seen from Fig 21. The lower and middle branches join up as the coupling gain is increased (for *K* = 250, 300), which leads to the appearance of a saddle-node in the bifurcation diagram of the system. Hence, for the second case, we studied the bifurcation diagram for interconnection gains *K* = 50, 250. In all bifurcation diagrams, the stability of equilibria has been determined by computing the eigenvalues of Jacobian matrix which is represented by

$$\begin{array}{c} \begin{array}{c} J=[\begin{array}{cc} J_{11} & J_{12}\\ J_{21} & J_{22} \end{array}] \end{array} \end{array}$$ (15)

where $J_{jj}=\left[J_{jj}^{1}J_{jj}^{2}\right]$ with

$$\begin{array}{c} \begin{array}{cc} J_{jj}^{1}= & [\begin{array}{cccc} 0 & 1 & 0 & 0\\ -{\zeta_{e}^{j}}^{2} & -2\zeta_{e}^{j} & \alpha_{e}^{j}\zeta_{e}^{j}h\left(v_{1}^{j}-v_{2}^{j}\right) & 0\\ 0 & 0 & 0 & 1\\ \alpha_{e}^{j}\zeta_{e}^{j}c_{ep}^{j}c_{pe}^{j}h\left(c_{pe}^{j}v_{0}^{j}\right) & 0 & -{\zeta_{e}^{j}}^{2} & -2\zeta_{e}^{j}\\ 0 & 0 & 0 & 0\\ \alpha_{i}^{j}\zeta_{i}^{j}c_{ip}^{j}c_{pi}^{j}h\left(c_{pi}^{j}v_{0}^{j}\right) & 0 & 0 & 0\\ 0 & 0 & 0 & 0\\ 0 & 0 & \alpha_{e}^{j}\zeta_{d}^{j}h\left(v_{1}^{j}-v_{2}^{j}\right) & 0 \end{array}],\\ J_{jj}^{2}= & [\begin{array}{cccc} 0 & 0 & 0 & 0\\ -\alpha_{e}^{j}\zeta_{e}^{j}h\left(v_{1}^{j}-v_{2}^{j}\right) & 0 & 0 & 0\\ 0 & 0 & 0 & 0\\ 0 & 0 & 0 & 0\\ 0 & 1 & 0 & 0\\ -{\zeta_{i}^{j}}^{2} & -2\zeta_{i}^{j} & 0 & 0\\ 0 & 0 & 0 & 1\\ -\alpha_{e}^{j}\zeta_{d}^{j}h\left(v_{1}^{j}-v_{2}^{j}\right) & 0 & -{\zeta_{d}^{j}}^{2} & -2\zeta_{d}^{j} \end{array}], \end{array} \end{array}$$ (16)

and $h\left(v\right)=\frac{2e_{0}r\;exp\left(r\left(v_{th}-v\right)\right)}{{\left(1+\text{exp}\left(r\left(v_{th}-v\right)\right)\right)}^{2}}$ for j = 1,2. Furthermore, the matrices *J*_*jl*_ for *j* ≠ *l* are defined as

$$\begin{array}{c} \begin{array}{c} J_{jl}=[\begin{array}{cccccccc} 0 & 0 & 0 & 0 & 0 & 0 & 0 & 0\\ 0 & 0 & 0 & 0 & 0 & 0 & 0 & 0\\ 0 & 0 & 0 & 0 & 0 & 0 & 0 & 0\\ 0 & 0 & 0 & 0 & 0 & 0 & \alpha_{e}^{j}\zeta_{e}^{j}K^{j,l} & 0\\ 0 & 0 & 0 & 0 & 0 & 0 & 0 & 0\\ 0 & 0 & 0 & 0 & 0 & 0 & 0 & 0\\ 0 & 0 & 0 & 0 & 0 & 0 & 0 & 0\\ 0 & 0 & 0 & 0 & 0 & 0 & 0 & 0 \end{array}]. \end{array} \end{array}$$ (17)

## D Estimation method

### D.1 Augmented model of a cortical region

The model of a cortical region Eq (1) can be written in the form

$$\begin{array}{c} \dot{x}=Ax+B\vec{\phi }(Cx), \end{array}$$ (18)

where $x\in R^{N_{x}}$ is a state vector representing the postsynaptic membrane potentials generated by each population synapse and their time derivatives. There are two states per synapse and *N*_*x*_ = 2*N*_*s*_ is the total number of states, where for *N*_*s*_ synaptic connections in the models the state vector is of the form

$$\begin{array}{c} x={\left[\begin{array}{ccccc} v_{1} & z_{1} & \ldots & v_{N_{s}} & z_{N_{s}} \end{array}\right]}^{⊤}. \end{array}$$

The matrix **A** encodes the dynamics induced by the membrane time constants. For *N*_*s*_ synapses, **A** has the block diagonal structure

$$\begin{array}{c} A=\text{diag}(\begin{array}{ccc} \Psi_{1} & \ldots & \Psi_{N_{s}} \end{array}), \end{array}$$

where the *n*^*th*^ synapse is described by

$$\begin{array}{c} \Psi_{n}=[\begin{array}{cc} 0 & 1\\ -\zeta_{n}^{2} & -2\zeta_{n} \end{array}]. \end{array}$$

The matrix of synaptic gains from internal inputs, **B**, has the diagonal form

$$\begin{array}{c} B=\text{diag}(\begin{array}{ccccc} 0 & \alpha_{1} & \ldots & 0 & \alpha_{N_{s}} \end{array}), \end{array}$$

The vector function *ϕ*(⋅) has the following form

$$\begin{array}{c} \vec{\phi }\left(Cx\right)={\left[\begin{array}{ccccccc} 0 & g\left(c_{2,:}x\right) & \ldots & 0 & g\left(c_{N_{x}-2,:}x\right) & 0 & u \end{array}\right]}^{⊤} \end{array}$$ (19)

The adjacency matrix, **C**, defines the connectivity structure of the model. It is a matrix of zeros or ones that specifies all the connections between the cell population types (excluding external inputs) that has the block structure

$$\begin{array}{c} C=[\begin{array}{ccccc} 0 & 0 & \ldots & 0 & 0\\ c_{2,1} & 0 & & c_{2,N_{x}-1} & 0\\ \vdots & & \ddots & & \vdots \\ 0 & 0 & & 0 & 0\\ c_{N_{x},1} & 0 & & c_{N_{x},N_{x}-1} & 0 \end{array}]. \end{array}$$

For example, if the PSPs from synapses 1 and 2 are summed and transformed by the sigmoid to give the input firing rate to synapse *n*, then row 2*n* of **C** with have the form

$$\begin{array}{c} c_{2n,:}=[\begin{array}{ccccccccc} 1 & 0 & 1 & 0 & 0 & 0 & \ldots & 0 & 0 \end{array}]. \end{array}$$

It is necessary to discretize the model to numerically integrate the equations and run simulations. The discrete time version of the model is

$$\begin{array}{c} x_{t+1}=A^{\delta }x_{t}+B^{\delta }\vec{\phi }(Cx_{t})+w_{t}. \end{array}$$ (20)

The matrices **A**^*δ*^ and **B**^*δ*^ are discrete time versions of **A** and **B**, respectively, and are defined in [28]. For ease of notation, we shall abbreviate increments or decrements in time (by the integration time step) by *t* + 1 or *t* − 1, respectively. The additional term **w**_*t*_ captures uncertainty in the neural mass model for estimation purposes ($w_{t}∼N(0,Q)\in R^{N_{x}}$).

The neural mass model is mapped to electrophysiological measurements by the observation equation

$$\begin{array}{cc} y_{t}= & Hx_{t}+v_{t}, \end{array}$$ (21)

where $H\in R^{N_{x}\times N_{y}}$ is the observation matrix, $v∼N(0,R)\in R^{N_{y}}$ is the observation noise, *N*_*x*_ is the number of states, and *N*_*y*_ is the number of observations.

### D.2 Re-parametrization for model for inversion

To estimate the input within our framework, we assume that it is varying on a time scale much slower than the state variables (*v* and *z*). Following this assumption we can reduce the model dimension since

$$\begin{array}{c} v_{p3}=\text{constant}=\tilde{u}\approx \alpha_{e}\xi_{e}\frac{u}{\xi_{e}^{2}}, \end{array}$$ (22)

in the steady state limit. A further modification for model inversion induces a new parameter, λ, to deal with DC offsets on the EEG signals due to electrode-tissue interactions. The offset parameter is added to the post-synaptic potential at the excitatory to pyramidal connection Eq (1),

$$\begin{array}{c} {\dot{v}}_{p1}=z_{p1}+\lambda , \end{array}$$ (23)

but removed from it where it feeds back to the system in the sigmoidal activation function. This way the system dynamics are unaffected by this addition, but the observation is offset by λ (since *v*_*p*1_ contributes to the EEG). The additional parameter enables us to estimate a slowly (with respect to the sate variables) changing DC offset in real data. We also modify the form of the activation function *g*(⋅) to

$$\begin{array}{c} g\left(v\right)\approx \tilde{g}\left(v\right)=\frac{1}{2}(\text{erf}(\frac{v-v_{0}}{\varsigma })+1) \end{array}$$ (24)

where *ς* = 1.699/*r*. The function $\tilde{g}\left(\cdot \right)$ enables precise propagation of Gaussian distribution through time in the estimation method. It only differs from *g*(⋅) slightly at the turning points of the sigmoid and does not change the dynamics of the system significantly.

The modified vectorized activation function has the following form

$$\begin{array}{c} \tilde{\vec{\phi }}\left(Cx\right)={\left[\begin{array}{ccccccc} 0 & \tilde{g}\left(c_{2,:}x+\tilde{u}-\lambda \right) & 0 & \tilde{g}\left(c_{4,:}x+\tilde{u}-\lambda \right) & \ldots & 0 & \tilde{g}\left(c_{{\tilde{N}}_{x}-2,:}x\right) \end{array}\right]}^{⊤} \end{array}$$ (25)

where ${\tilde{N}}_{x}=N_{x}-2$ (from the input modification).

Any neural mass model with an arbitrary number of populations can be written in the form described above, including the model of the two coupled regions that we employ in this paper. It is straight forward to construct the matrices **A**, **B** and **C**, therefore for the sake of conciseness, we leave the basic form of the state-space model here.

### D.3 Augmentation for model for inversion

In order to perform online joint state and parameter estimation we augment the model and concatenate the inputs and measurement offsets to the state vector. To define to the augmented model we first define a vector of parameters as

$$\begin{array}{c} \theta ={\left[\begin{array}{cccc} u^{a} & u^{b} & \lambda^{a} & \lambda^{b} \end{array}\right]}^{⊤}. \end{array}$$

The trivial dynamics for the parameter are model as

$$\begin{array}{c} \dot{\theta }=0 \end{array}$$ (26)

or in discrete time as

$$\begin{array}{c} \theta_{t+1}=\theta_{t}. \end{array}$$ (27)

The state vector **x** and the parameter vector ***θ*** are concatenated to form the augmented state vector

$$\begin{array}{c} \xi ={\left[\begin{array}{cc} x^{T} & \theta^{T} \end{array}\right]}^{⊤}. \end{array}$$ (28)

Our augmented state-space model is

$$\begin{array}{c} \xi_{t}=A_{\theta }\xi_{t-1}+B_{\theta }\phi \left(C_{\theta }\xi_{t-1}\right)+w_{t-1} \end{array}$$ (29)

where $w_{t}∼N(0,Q)$. The state vector $\xi \in R^{N_{\xi }\times 1}$ and matrices **A**_*θ*_, **B**_*θ*_, and **C**_*θ*_ are $\in R^{N_{\xi }\times N_{\xi }}$ and have the form

$$\begin{array}{c} A_{\theta }=[\begin{array}{cc} A_{\delta } & 0\\ 0 & I \end{array}],B_{\theta }=[\begin{array}{cc} B & 0\\ 0 & 0 \end{array}],C_{\theta }=[\begin{array}{cc} C & 0\\ 0 & 0 \end{array}]. \end{array}$$ (30)

To make the next step a little easier we will simplify the notation by dropping the subscript *θ* on the system matrices and abbreviate the activation function giving

$$\begin{array}{c} \xi_{t}=A\xi_{t-1}+B\phi \left(C\xi_{t-1}\right)+w_{t-1}. \end{array}$$ (31)

### D.4 A filter for the population model

The filter provides an estimate of the most likely sequences of states, ${\hat{\xi }}_{t}^{+}$, and the associated error covariances, ${\hat{P}}_{t}^{+}$, given (uncertain) knowledge of the biophysics and anatomy of the brain regions of interest combined with the noisy EEG measurements, **y**_*t*_. The method is based on the Kalman filter [33], but falls in the category of an assumed density filter (using a Gaussian prior). The optimal state estimates can be formally stated using the expectations

$$\begin{array}{cc} {\hat{\xi }}_{t}^{+}= & E[\xi_{t}|y_{1},y_{2},\ldots ,y_{t}] \end{array}$$ (32)

$$\begin{array}{c} {\hat{P}}_{t}^{+}=E\left[\left(\xi_{t}-{\hat{\xi }}_{t}^{+}\right){\left(\xi_{t}-{\hat{\xi }}_{t}^{+}\right)}^{⊤}\right], \end{array}$$ (33)

which are known as the a posteriori state estimate and state estimate covariance, respectively. The a posteriori state estimate is computed by correcting the a priori state estimate, which is a prediction though our model and defined as [28]

$$\begin{array}{ll} {\hat{\xi }}_{t}^{-} & =E\left[\xi_{t}|y_{1},y_{2},\ldots ,y_{t-1}\right]\\ & =E\left[A\xi_{t-1}+B\phi \left(\xi_{t-1}\right)+w_{t}\right]\\ & =A{\hat{\xi }}_{t-1}^{+}+B{\hat{\phi }}_{t-1}, \end{array}$$ (34)

where the vectors (note the square root is element-wise and ∘ is the Hadamard product)

$$\begin{array}{ll} {\hat{\phi }}_{t-1} & =\frac{1}{2}\left(\text{erf}\left(\beta \circ \sigma^{-1/2}\right)+1\right)\\ \beta_{t} & =C{\hat{\xi }}_{t-1}^{+}-v_{0}\\ \sigma_{t} & =2\left(\text{diag}\left(CP_{t-1}^{+}C^{⊤}\right)+\varsigma^{2}\right). \end{array}$$ (35)

The a posteriori state estimate is calculated using a weighted difference between an uncertain prediction of the observations (EEG) and the actual noisy measurements

$${\hat{\xi }}_{t}^{+}={\hat{\xi }}_{t}^{-}+K_{t}\underset{\text{EEG}\;\;\text{prediction}\;\;\text{error}}{\underset{︸}{\left(y_{t}-H{\hat{\xi }}_{t}^{-}\right)}}.$$ (36)

The weighting to correct the a priori augmented state estimate, $K_{t}$, is known as the Kalman gain. The Kalman gain is computed from the confidence in a prediction of the augmented state and the noisy measurement model by

$$\begin{array}{c} K_{t}={\hat{P}}_{t}^{-}H^{⊤}{\left(H{\hat{P}}_{t}^{-}H^{⊤}+\kappa_{t}R\right)}^{-1}, \end{array}$$ (37)

where *κ*_*t*_ is an annealing parameter. The annealing schedule is

$$\begin{array}{c} \kappa_{t}=\kappa_{0}^{\frac{t_{a}-t}{t_{a}-1}} \end{array}$$ (38)

and *κ*_0_ is a larger number. Following this schedule the annealing parameter will decrease from *κ*_0_ to 1 following a geometric series. When the annealing parameter is high, the Kalman gain is small and the measurements are not full utilized. The annealing has the effect of slowly introducing corrections from the measurements on initialization, avoiding taking large steps towards local minima when our initial uncertainty is high. The a priori state estimate error covariance is

$$\begin{array}{ll} {\hat{P}}_{t}^{-} & =E\left[\left(\xi_{t}-{\hat{\xi }}_{t}^{-}\right){\left(\xi_{t}-{\hat{\xi }}_{t}^{-}\right)}^{⊤}\right]\\ & =E\left[\left(A\xi_{t-1}+B\phi \left(C\xi_{t-1}\right)+w_{t-1}-\left(A{\hat{\xi }}_{t-1}^{+}+B{\hat{\phi }}_{t-1}\right)\right){\left(\cdot \right)}^{⊤}\right]\\ & =A{\hat{P}}_{t-1}^{+}A^{⊤}+BE\left[\phi \left(\xi_{t-1}\right)\phi^{⊤}\left(\xi_{t-1}\right)\right]B^{⊤}+Q-B{\hat{\phi }}_{t-1}{\hat{\phi }}_{t-1}^{⊤}B^{⊤}+\Phi_{t-1}+\Phi_{t-1}^{⊤}, \end{array}$$ (39)

where

$$\begin{array}{ll} \Phi_{t-1} & =AE\left[\xi_{t-1}\phi^{⊤}\left(C\xi_{t-1}\right)\right]B^{⊤}-A{\hat{\xi }}_{t-1}^{+}E\left[\phi^{⊤}\left(C\xi_{t-1}\right)\right]B^{⊤}\\ & =A\left(P_{t-1}^{+}C^{⊤}\circ 1\times \Lambda^{⊤}\right)B^{⊤} \end{array}$$ (40)

$$\begin{array}{c} \Lambda ={\left(\pi \sigma \right)}^{-1/2}\text{exp}\left(-\beta \circ \beta \circ \sigma^{-1}\right). \end{array}$$ (41)

We can analytically calculate all the elements of ${\hat{P}}_{t}^{-}$ except for $E[\phi \left(\xi_{t-1}\right)\phi^{⊤}\left(\xi_{t-1}\right)]$, which is know to have no analytic solution. Nevertheless, we can compute a precise solution (to error of 10^−14^) as explained in [39]. The elements, indexed by *i* and *j*, of the matrix resulting from evaluating the expectation are equivalent to the probabilities of the bivariate Gaussians

$$\begin{array}{c} E{\left[\phi \left(C\xi_{t-1}\right)\phi^{⊤}\left(C\xi_{t-1}\right)\right]}_{ij}=P(x>0,y>0), \end{array}$$ (42)

where ${\left(x,y\right)}^{⊤}∼N\left(\mu ,\Sigma \right)$ and

$$\begin{array}{ll} \mu & =-{\left[{\left(C{\hat{\xi }}_{t-1}^{+}\right)}_{i},{\left(C{\hat{\xi }}_{t-1}^{+}\right)}_{j}\right]}^{⊤}\\ \Sigma & =\left[\begin{array}{cc} {\left(\text{diag}\left(C{\hat{P}}_{t-1}^{+}C^{⊤}\right)+\varsigma^{2}\right)}_{i} & {\left(C{\hat{P}}_{t-1}^{+}C^{⊤}\right)}_{ij}\\ {\left(C{\hat{P}}_{t-1}^{+}C^{⊤}\right)}_{ij} & {\left(\text{diag}\left(C{\hat{P}}_{t-1}^{+}C^{⊤}\right)+\varsigma^{2}\right)}_{j} \end{array}\right]. \end{array}$$

These probabilities can be computed easily in Matlab using, where each element is mvncdf (**0**, ***μ***, **Σ**).

For a linear observation function, the a posteriori covariance is then updated by using the Kalman gain to provide the correction

$$\begin{array}{c} {\hat{P}}_{t}^{+}=(I-K_{t}H){\hat{P}}_{t}^{-}. \end{array}$$ (43)

Practically, the actual state is not known so the Kalman filter must be initialized with the best guess for ${\hat{\xi }}_{0}^{+}$ and ${\hat{P}}_{0}^{+}$, which provides the a posteriori state estimate and state estimate covariance for time *t* = 0. The other parameters that must be initialized are **Q** and **R**.

### D.5 Filter initialization

This section provides the parameter values that were initialized prior to implementing the assumed density filter. Values are also provided in the code (https://github.com/pkaroly/Bifurcation-Estimation)

To initialize ${\hat{\xi }}_{0}^{+}$ and ${\hat{P}}_{0}^{+}$ we used the empirical mean and covariance of the states based on a forward simulation of the model.

The model and measurement noise, **Q** and **R** are given by

$$\begin{array}{c} Q=\{\begin{array}{ccccccc} \sigma_{v} & 0 & \ldots & 0 & 0 & 0 & 0\\ 0 & \sigma_{z} & \ldots & 0 & 0 & 0 & 0\\ & & & \ddots & & & \\ 0 & 0 & \ldots & \sigma_{v} & 0 & 0 & 0\\ 0 & 0 & \ldots & 0 & \sigma_{z} & 0 & 0\\ 0 & 0 & \ldots & 0 & 0 & \sigma_{u} & 0\\ 0 & 0 & \ldots & 0 & 0 & 0 & \sigma_{\lambda } \end{array}\} \end{array}$$ (44)

$$\begin{array}{c} R=\{\begin{array}{cc} \sigma_{y} & 0\\ 0 & \sigma_{y} \end{array}\} \end{array}$$ (45)

The terms *σ*_*v*_ and *σ*_*z*_ are the standard deviation of the model noise for the membrane potentials, *v* and derivatives, *z*. The terms *σ*_*u*_ and *σ*_λ_ correspond to the model noise of the input and DC offset. We did not explicitly include model uncertainty for the derivatives and input offset; however, we set *σ*_*z*_ and *σ*_λ_ to small positive numbers for numerical stability of the filter.

$$\begin{array}{cc} \sigma_{v} & =1\times 10^{-5}\;V\\ \sigma_{z} & =1\times 10^{-8}\;V\\ \sigma_{u} & =1\times 10^{-5}\;V\\ \sigma_{\lambda } & =1\times 10^{-8}\;V \end{array}$$ (46)

The term *σ*_*y*_ = 1 × 10^−4^ *V* is the standard deviation of the measurement noise.

The values of *σ*_*v*_, *σ*_*u*_ and *σ*_*y*_ reflect the relative certainty in the model as opposed to the data. Practically, these values require some tuning to achieve filter stability, with a balance between perfectly tracking the recorded ECoG (overfitting), versus ignoring the data. For a more thorough discussion of the effects of tuned parameters on the estimation accuracy the reader is referred to [28].
